# Multifunctional pH-responsive HA-c-FZ1 hydrogel presents a promising therapeutic strategy for diabetic skin wounds

**DOI:** 10.1093/burnst/tkag046

**Published:** 2026-07-05

**Authors:** Zhe Fu, Jingyu Jiang, Yutong Wu, Zhaoxun Xiao, Yunhan Su, Hao Zhu, Shaowei Chen, Xinhe Shen, Xinyue Yang, Yunjian Luo, Tuanqing Li, Shibo Wang, Ying Wang, Xinwang Yang, Ying Yang

**Affiliations:** Department of Endocrinology, Affiliated Hospital of Yunnan University, No. 176 Qingnian Road, Wuhua District, Kunming, Yunnan 650021, China; Key Laboratory of Skin and Mucosal Injury Repair, Regeneration and Active Peptides of Yunnan Provincial Department of Education, Regenerative Medicine Research Center, Faculty of Basic Medical Science, Kunming Medical University, No. 1168 Chunrong West Road, Chenggong District, Kunming, Yunnan 650500, China; Department of Endocrinology, Affiliated Hospital of Yunnan University, No. 176 Qingnian Road, Wuhua District, Kunming, Yunnan 650021, China; Key Laboratory of Skin and Mucosal Injury Repair, Regeneration and Active Peptides of Yunnan Provincial Department of Education, Regenerative Medicine Research Center, Faculty of Basic Medical Science, Kunming Medical University, No. 1168 Chunrong West Road, Chenggong District, Kunming, Yunnan 650500, China; Key Laboratory of Skin and Mucosal Injury Repair, Regeneration and Active Peptides of Yunnan Provincial Department of Education, Regenerative Medicine Research Center, Faculty of Basic Medical Science, Kunming Medical University, No. 1168 Chunrong West Road, Chenggong District, Kunming, Yunnan 650500, China; School of Nursing and Health, Nanfang College Guangzhou, No. 882 Wenquan Avenue, Conghua District, Guangzhou, Guangdong 510970, China; Key Laboratory of Skin and Mucosal Injury Repair, Regeneration and Active Peptides of Yunnan Provincial Department of Education, Regenerative Medicine Research Center, Faculty of Basic Medical Science, Kunming Medical University, No. 1168 Chunrong West Road, Chenggong District, Kunming, Yunnan 650500, China; Key Laboratory of Skin and Mucosal Injury Repair, Regeneration and Active Peptides of Yunnan Provincial Department of Education, Regenerative Medicine Research Center, Faculty of Basic Medical Science, Kunming Medical University, No. 1168 Chunrong West Road, Chenggong District, Kunming, Yunnan 650500, China; Key Laboratory of Skin and Mucosal Injury Repair, Regeneration and Active Peptides of Yunnan Provincial Department of Education, Regenerative Medicine Research Center, Faculty of Basic Medical Science, Kunming Medical University, No. 1168 Chunrong West Road, Chenggong District, Kunming, Yunnan 650500, China; Key Laboratory of Skin and Mucosal Injury Repair, Regeneration and Active Peptides of Yunnan Provincial Department of Education, Regenerative Medicine Research Center, Faculty of Basic Medical Science, Kunming Medical University, No. 1168 Chunrong West Road, Chenggong District, Kunming, Yunnan 650500, China; Key Laboratory of Skin and Mucosal Injury Repair, Regeneration and Active Peptides of Yunnan Provincial Department of Education, Regenerative Medicine Research Center, Faculty of Basic Medical Science, Kunming Medical University, No. 1168 Chunrong West Road, Chenggong District, Kunming, Yunnan 650500, China; Key Laboratory of Skin and Mucosal Injury Repair, Regeneration and Active Peptides of Yunnan Provincial Department of Education, Regenerative Medicine Research Center, Faculty of Basic Medical Science, Kunming Medical University, No. 1168 Chunrong West Road, Chenggong District, Kunming, Yunnan 650500, China; Key Laboratory of Skin and Mucosal Injury Repair, Regeneration and Active Peptides of Yunnan Provincial Department of Education, Regenerative Medicine Research Center, Faculty of Basic Medical Science, Kunming Medical University, No. 1168 Chunrong West Road, Chenggong District, Kunming, Yunnan 650500, China; Key Laboratory of Skin and Mucosal Injury Repair, Regeneration and Active Peptides of Yunnan Provincial Department of Education, Regenerative Medicine Research Center, Faculty of Basic Medical Science, Kunming Medical University, No. 1168 Chunrong West Road, Chenggong District, Kunming, Yunnan 650500, China; Key Laboratory of Chemistry in Ethnic Medicinal Resources and Key Laboratory of Natural Products Synthetic Biology of Ethnic Medicinal Endophytes, State Ethnic Affairs Commission and Ministry of Education, School of Ethnic Medicine, Yunnan Minzu University, No. 2929 Yuehua Road, Chenggong District, Kunming, Yunnan 650504, China; Key Laboratory of Skin and Mucosal Injury Repair, Regeneration and Active Peptides of Yunnan Provincial Department of Education, Regenerative Medicine Research Center, Faculty of Basic Medical Science, Kunming Medical University, No. 1168 Chunrong West Road, Chenggong District, Kunming, Yunnan 650500, China; Yunnan Characteristic Plant Extraction Laboratory Co., Ltd., No. 53 Keyi Road, High-tech Industrial Development Zone, Kunming, Yunnan 650106, China; Department of Endocrinology, Affiliated Hospital of Yunnan University, No. 176 Qingnian Road, Wuhua District, Kunming, Yunnan 650021, China

**Keywords:** Diabetic foot ulcers, Bioactive peptide FZ1, Hyaluronic acid hydrogel, Photo-crosslinking, Anti-inflammation, Oxidative stress

## Abstract

**Background:**

Diabetic foot ulcers remain a major clinical challenge, driven by the convergent effects of microbial infection, chronic inflammation, oxidative stress, and inadequate neovascularization that collectively disrupt tissue repair. Although the integrin αvβ3 agonist peptide FZ1 exhibits promising pro-healing activity, limited delivery control and insufficient functional versatility have restricted its clinical translation. To overcome these constraints, a pH-responsive hyaluronic acid (HA)-based hydrogel, hyaluronic acid conjugated with FZ1 peptide (HA-c-FZ1), was developed to enable intelligent acidity-triggered peptide release while integrating antimicrobial activity, reactive oxygen species (ROS) scavenging, and inflammation control within a single wound dressing platform for diabetic wound repair.

**Methods:**

Photo-crosslinkable oxidized methacrylated HA was synthesized and covalently linked to FZ1 through a pH-labile Schiff base bond, yielding HA-c-FZ1 hydrogel at three concentrations (2%, 3%, and 4%). HA-c-FZ1 hydrogel morphology, swelling behavior, mechanical properties, peptide-loading efficiency, pH-responsive release, antioxidant activity, antibacterial activity, biocompatibility, and anti-inflammatory effects were systematically evaluated *in vitro*. Therapeutic efficacy was then assessed in a diabetic mouse model with infected full-thickness skin wounds through wound closure analysis, histological staining, immunofluorescence detection of macrophage polarization markers (inducible nitric oxide synthase and arginase) and angiogenic markers (alpha-smooth muscle actin and vascular endothelial growth factor), and laser speckle imaging of blood perfusion. Transcriptomic profiling, RT-qPCR, and western blotting were performed to define the molecular mechanisms underlying HA-c-FZ1-mediated wound repair.

**Results:**

HA-c-FZ1 hydrogel formed a homogeneous porous hydrogel network with predictable swelling behavior, tunable mechanical performance, high FZ1 loading efficiency, and accelerated peptide release under acidic conditions. *In vitro*, HA-c-FZ1 hydrogel promoted keratinocyte proliferation and migration, reduced ROS accumulation, and inhibited *Staphylococcus aureus* and *Escherichia coli* survival. In infected diabetic wounds, HA-c-FZ1 hydrogel suppressed bacterial burden and accelerated wound closure, accompanied by enhanced re-epithelialization, collagen deposition, angiogenesis, and local blood perfusion. Mechanistic analyses indicated that HA-c-FZ1 hydrogel attenuated nuclear factor kappa B-associated inflammatory signaling, promoted M2 macrophage polarization, and activated focal adhesion kinase–protein kinase B-dependent angiogenic responses.

**Conclusions:**

HA-c-FZ1 functions as a pH-responsive hydrogel dressing that combines peptide delivery with antimicrobial, redox-regulatory, immunomodulatory, and pro-angiogenic functions. These findings position it as a therapy for chronic diabetic wound repair by simultaneously addressing infection, inflammation, oxidative stress, and vascular regeneration.

## Graphical Abstract

**Graphical Abstract:**
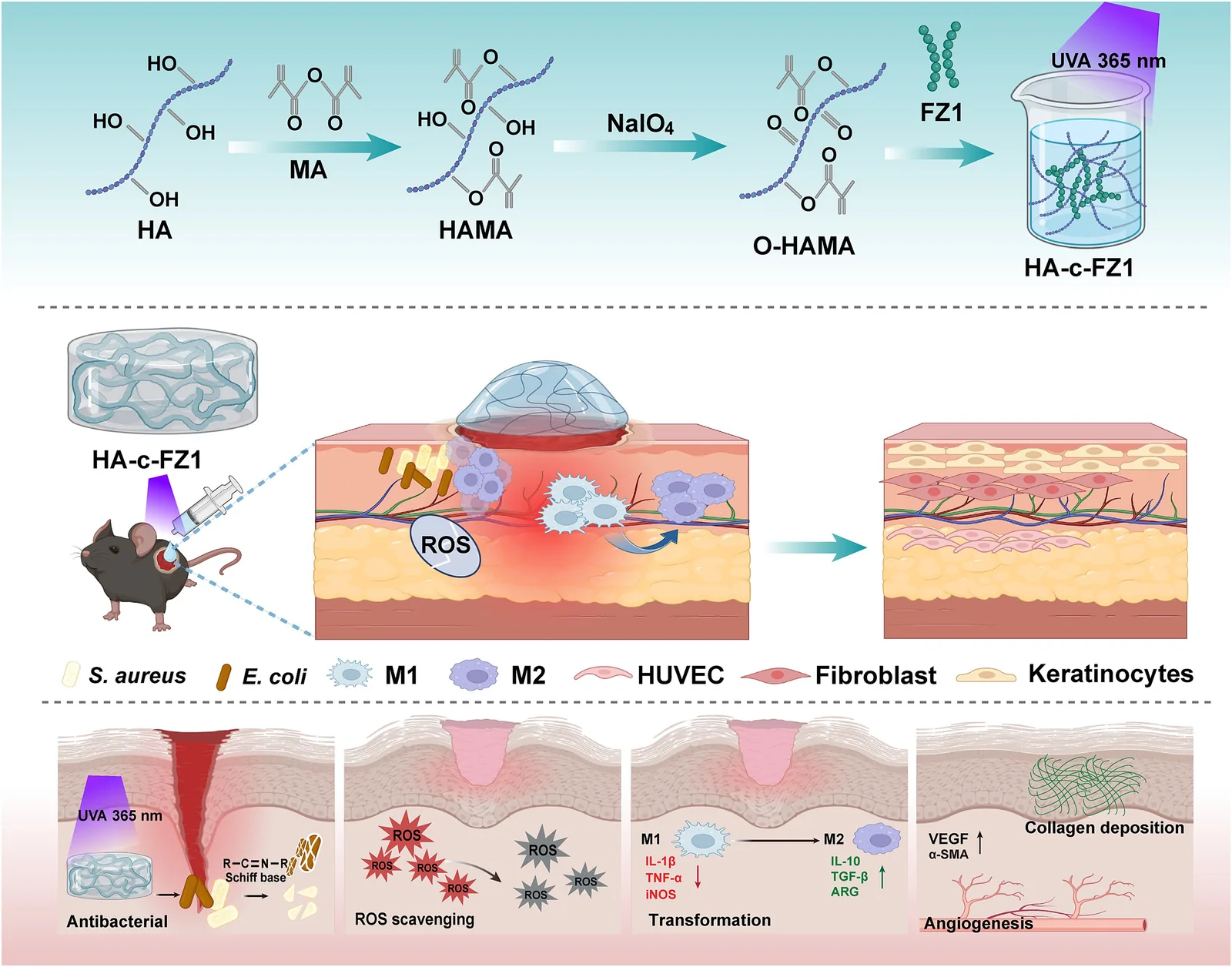

## Highlights

- A multifunctional pH-responsive hydrogel (HA-c-FZ1), engineered by conjugating the FZ1 peptide to a photo-crosslinkable HA backbone, enables intelligent acid-triggered release of FZ1 and shows high drug loading efficiency.
- A pH-responsive HA-c-FZ1 hydrogel integrates FZ1 peptide delivery with antimicrobial, antioxidant, and anti-inflammatory functions, thereby accelerating infected diabetic wound healing by promoting re-epithelialization, collagen deposition, and angiogenesis.
- HA-c-FZ1 hydrogel exerts its effects by inhibiting nuclear factor kappa B signaling, promoting M2 macrophage polarization, and activating the focal adhesion kinase–protein kinase B pathway.

## Background

Diabetic foot ulcers (DFUs) are among the most disabling complications of diabetes, with an estimated lifetime burden affecting 19%–34% of patients [1]. Clinical progression is frequently severe, with ~20% of cases ultimately requiring amputation, which is associated with a 10-year mortality rate approaching 70% [2]. Defective healing reflects a highly dysregulated wound microenvironment in which oxidative stress, impaired angiogenesis, chronic inflammation, and biofilm-associated infection collectively arrest the transition from tissue injury to productive repair [3]. While standard clinical interventions, including glycemic management and debridement, can provide partial metabolic and local wound control, >50% of patients with DFUs fail to achieve complete epithelialization within 20 weeks [4]. This therapeutic gap arises in part from the limited capacity of conventional treatments to recalibrate the multiple, interacting pathologic processes that sustain chronic wounds. Stem cell-based therapies and bioengineered dressings have expanded the therapeutic landscape; however, clinical translation remains constrained by poor cell survival, inconsistent standardization, and insufficient capacity for adaptive, multi-pathway targeting [5, 6]. Therapeutic platforms that can respond dynamically to the chronic wound microenvironment while coordinating multiple functions remain a critical unmet need.

Amphibian-derived bioactive peptides are an attractive resource for regenerative medicine. They were structurally diverse, have low toxicity, and show high target specificity [7–10]. Several wound-healing peptides from amphibians have been identified in earlier work, including RL-QN15, OA-RD17, Andersonin-W1, and OA-GL17d [11–14]. Cy_RL-QN15_ (CQFHYMC), a stable cyclic heptapeptide containing the conserved C-X5-C domain, was produced by structural optimization of RL-QN15 and showed stronger activity in chronic wound repair [15]. Based on this result, the C-X5-C scaffold could serve as a compact module for designing regenerative peptides. This motif-centered design guided the structural refinement of Odorlanin-C1, which produced FZ1 (CKLSNDC), a cyclic heptapeptide whose sequence fits the C-X5-C scaffold. Its therapeutic efficacy in chronic wound models exceeded that of previous peptide candidates. FZ1 was identified as the first known high-affinity peptide agonist of integrin αvβ3, which promotes diabetic wound repair through the focal adhesion kinase (FAK)/PI3K-protein kinase B (AKT)/extracellular signal-regulated kinase (ERK) pathway and VEGFC upregulation [16]. FZ1 exhibited potent pro-repair activity, but its therapeutic use is limited by an ineffective delivery platform and limited functional versatility in chronic diabetic wounds.

Hyaluronic acid (HA) is a naturally occurring polysaccharide and a core biomaterial for wound repair. It has tunable mechanical properties, chemical versatility, and pro-regenerative activity [17, 18]. HA-based matrices keep the wound hydrated and provide a scaffold for supporting cell migration, matrix deposition, and tissue repair [19]. The HA backbone has many reactive chemical groups for covalent coupling with therapeutic cargos, enabling stable peptide immobilization, protection, and controlled release in hydrogel networks [20, 21]. These dynamic covalent HA hydrogels made with oxidized chondroitin sulfate or ε-polylysine as crosslinkers address key limitations of free peptides such as rapid enzymatic degradation, poor retention, and short half-life [22–24]. These systems support wound healing through effects on microbial control, inflammation, vascular regeneration, and ECM remodeling [25–28]. Static HA hydrogels are poorly suited to diabetic wounds because they cannot adapt to a shifting environment where acidosis, oxidative stress, protease activity, and inflammation fluctuate over time. This limitation has driven the development of stimulus-responsive hydrogel platforms that convert pathological signals into controlled therapeutic release [29, 30]. Incorporation of pH-responsive polymers, reactive oxygen species (ROS)-labile thioether linkers, or other labile motifs enables spatiotemporally controlled drug release in response to pathological cues within the wound microenvironment, including reduced pH, elevated ROS, and aberrant protease activity [31, 32]. For example, a metformin-loaded chitosan/oxidized HA hydrogel achieved a cumulative drug release rate of up to 80% within 72 h at pH 5.5, markedly exceeding release under physiological pH 7.4 conditions [33]. Such adaptive systems improve therapeutic precision by restricting release to pathological wound sites, increasing drug bioavailability, reducing off-target exposure, and aligning antimicrobial, immunomodulatory, pro-angiogenic, and matrix-remodeling activities with the sequential demands of inflammation, proliferation, and tissue remodeling [34, 35].

In this research, a multifunctional pH-responsive hydrogel delivery platform (hyaluronic acid conjugated with FZ1 peptide; HA-c-FZ1 hydrogel) was constructed from high-molecular-weight HA for chronic skin wound treatment. The system was designed to couple two complementary features: an acid-labile Schiff base linkage that covalently anchors the pro-healing peptide FZ1 and enables condition-triggered release within the acidic wound milieu, and a methacrylated HA framework that supports rapid photo-crosslinking and *in situ* formation of a stable, conformable dressing. This integrated architecture was intended to provide spatiotemporal control over peptide delivery while simultaneously modulating key barriers to diabetic wound repair, including infection, excessive inflammation, oxidative stress, and impaired angiogenesis. Physicochemical and functional characterization demonstrated that the HA-c-FZ1 hydrogel possessed suitable mechanical strength, regulated swelling behavior, sustained and pH-sensitive FZ1 release, efficient ROS scavenging, broad-spectrum antimicrobial activity, and favorable biocompatibility. In a subsequent diabetic wound model, HA-c-FZ1 hydrogel further promoted macrophage polarization toward an anti-inflammatory phenotype, enhanced collagen deposition, and stimulated angiogenesis, collectively accelerating tissue repair. These findings established an adaptive hydrogel strategy for translating amphibian-derived pro-regenerative peptides into multifunctional biomaterials for improved management of chronic skin wounds.

## Methods

### Preparation of photo-crosslinked HA-c-FZ1 hydrogel

The FZ1 peptide (sequence CKLSNDC, cyclized *via* a Cys1-Cys7 disulfide bond) was synthesized by solid-phase methods (Wuhan Tiande Biotechnology Co., Ltd.). Its purity exceeded 95%, as confirmed by HPLC and MS. Batch-to-batch consistency was verified across three independent batches, with no significant variation in activity or hydrogel performance. HA-c-FZ1 hydrogel was photo-crosslinked using modified protocols from previous reports [36–39]. Briefly, oxidized methacrylated hyaluronic acid (O-HAMA) was prepared from high-molecular-weight HA (1500 to 2500 kDa) by sequential methacrylation and oxidation. Ultrapure water (100 mL) was cooled in an ice bath. HA (2 g) was then dissolved in ultrapure water. NaOH (5 mmol/L, 3 mL) and methacrylic anhydride (6 mL) were added. The reaction was kept at 0 °C for 24 h. The product was dialyzed against ultrapure water (MWCO 3500 Da) for 4 days and lyophilized to obtain HAMA. HAMA was oxidized with NaIO_4_ at room temperature for 6 h. Ethylene glycol was then added to stop the reaction. The product was then dialyzed and lyophilized to obtain O-HAMA. For hydrogel preparation, FZ1 (10 nM) and LAP photoinitiator were mixed with O-HAMA (20, 30, and 40 mg portions), then vortexed until homogeneous. The mixture was exposed to 365 nm ultraviolet A (UVA) irradiation for 10 s to trigger crosslinking. Hydrogels were labeled as 2%, 3%, and 4% (w/v) HA-c-FZ1 based on O-HAMA concentration [40].

### Structural characterization and performance evaluation

Hydrogel microstructure was visualized by field-emission scanning electron microscopy (FE-SEM, Zeiss Sigma 360), and pore size was quantified using ImageJ software. Chemical functional groups were characterized by attenuated total reflectance Fourier-transform infrared spectroscopy (ATR-FTIR, Thermo Fisher Scientific Nicolet iS20) [41]. Structural characterization was further performed by ^1^H nuclear magnetic resonance (NMR) spectroscopy using a Bruker Avance NEO 600 MHz spectrometer [42]. Degrees of substitution (DS) for methacrylate and aldehyde groups were calculated from the corresponding NMR spectra.

An ethanol displacement method was used to measure porosity [43]. Hydrogel samples of known volume (V1) were placed in excess anhydrous ethanol (V2) until fully infiltrated. The displaced volume (V3) was then recorded, and porosity was derived as ((V1 − V3)/(V2 − V3)) × 100%. Lyophilized hydrogel samples were weighed for initial dry weight (We) and immersed in phosphate-buffered saline (PBS; pH 7.4) at 37°C to evaluate swelling behavior [44]. At predetermined intervals, samples were removed, surface liquid was gently blotted, and the swollen weight (W_t_) was recorded. The swelling ratio was calculated as [(W_t_ − W_e_)/W_e_] × 100%. Mechanical performance was assessed using a universal testing machine at a constant crosshead speed of 2 mm/min, and stress–strain curves were generated from the mechanical testing data [39].

### FZ1 loading content and efficiency

FZ1 loading in HA-c-FZ1 hydrogel was measured by a mass-balance method [45]. After photo-crosslinking, the mixture was centrifuged for 10 min. The supernatant was collected, and free FZ1 was quantified by HPLC against a standard curve. The unbound FZ1 mass (*M*_FZ1, free_) was calculated from this concentration, and the incorporated FZ1 (*M*_FZ1, in gel_) was derived by subtracting *M*_FZ1_, free from the total feeding mass (*M*_FZ1, feed_). The centrifuged hydrogels were lyophilized to constant weight for dry mass (*M*_gel, dry_) determination. Drug loading content (DLC) was calculated as (*M*_FZ1, in gel_/*M*_gel, dry_) × 100%. The loading efficiency (LE) was calculated as (*M*_FZ1, in gel_/*M*_FZ1, feed_) × 100%.

### Sustained release of FZ1 from HA-c-FZ1 hydrogel under different potential of hydrogen conditions

pH-responsive FZ1 release from HA-c-FZ1 hydrogel was evaluated using a dialysis-based approach [46]. Briefly, 2%, 3%, and 4% HA-c-FZ1 hydrogel formulations were sealed in dialysis bags with a molecular weight cutoff of 3500 Da and incubated in PBS at pH 5.5, 6.5, or 7.4 at 37 °C. At predetermined time points, the released FZ1 in the dialysate was quantified by measuring absorbance at 220 nm. Cumulative release percentages were then calculated for each formulation and pH condition.

### Preparation of hydrogel extracts

Hydrogel extracts were prepared according to previously established methods [47]. Briefly, 2%, 3%, and 4% HA-c-FZ1 hydrogel were separately immersed in Dulbecco’s Modified Eagle Medium (DMEM) at 0.1 g/mL and extracted with shaking at 120 r/min for 24 h in a 37 °C shaker. The resulting extracts were filtered through sterile syringe filters (0.45 μm) to obtain sterile hydrogel extracts for subsequent experiments.

### Bioadhesive properties

Bioadhesive performance was evaluated by a lap-shear test using fresh porcine skin [48]. Each hydrogel sample was applied to the defined lap-shear contact area, and standard shear testing was performed using a universal testing machine (MTS Industrial, CMT6104) in tensile mode at a constant shear rate of 2 mm/min, with data recorded throughout the test.

### Biocompatibility evaluation

Cellular biocompatibility was assessed by live/dead staining according to previously described protocols [49, 50]. HaCaT keratinocytes were resuspended in 2%, 3%, or 4% HA-c-FZ1 hydrogel extracts and seeded in 12-well plates at a density of 5 × 10^5^ cells/well. Cells were stained using a calcein acetoxymethyl/propidium iodide (calcein-AM/PI) kit (Beyotime, Shanghai, China) following the provided instructions, and imaged by confocal microscopy (Axio Observer Z1, Zeiss, Germany) [51].

### Cell proliferation and scratch wound healing assays

Cell viability was measured in HaCaT cells, human skin fibroblasts (HSFs), RAW264.7 macrophages, and human umbilical vein endothelial cells (HUVECs). Cells were suspended in hydrogel extracts of 2%, 3%, and 4% HA-c-FZ1 and seeded in 96-well plates at 9 × 10^3^ cells/well, then cultured at 37 °C for 24 h. Viability was quantified by the MTS assay (CellTiter 96 AQueous, Promega) at 490 nm. To assess scratch wound healing, 1 × 10^5^ HaCaT cells per well were seeded in 24-well plates and grown to 80% confluence. A sterile 200 μL pipette tip was used to create linear scratches. Cells then received 450 μL of serum-free DMEM with 50 μL of hydrogel extract. Migration was imaged at 0, 12, and 24 h under an inverted microscope, and wound closure was analyzed with ImageJ [52–54].

### *In vitro* antioxidant activity evaluation of HA-c-FZ1

The antioxidant activity of HA-c-FZ1 hydrogel was first evaluated by measuring intracellular ROS levels in HUVECs. Cells were seeded in 12-well plates at a density of 2 × 10^5^ cells/well and cultured to 70% confluence. Cells were then treated with 2%, 3%, or 4% hydrogel extracts and co-incubated with 20 μM H_2_O_2_ for 12 h. ROS levels were quantified using a commercial detection kit (Solarbio, China). Dichlorofluorescein (DCF) fluorescence was imaged by confocal microscopy (Axio Observer Z1, Zeiss) and quantified using ImageJ (NIH, USA) [55]. Radical scavenging activity was further assessed using ABTS^+^ [2,2′-azino-bis(3-ethylbenzothiazoline-6-sulfonic acid)] cation radicals and DPPH (1,1-diphenyl-2-picrylhydrazyl) radicals [56–58]. For ABTS^+^ scavenging, hydrogel extracts (50 μL) were mixed with ABTS^+^ solution (50 μL), incubated in the dark at room temperature for 15 min, and analyzed by absorbance measurement at 450 nm. For the DPPH assay, hydrogel extracts (10 μL) were mixed with DPPH solution (150 μL), incubated in the dark at room temperature for 30 min, and analyzed by absorbance measurement at 517 nm. Radical scavenging rates were calculated using standard formulas [59, 60].

### Hemocompatibility evaluation

Hemocompatibility of HA-c-FZ1 hydrogel was assessed using a standard hemolysis assay. Briefly, mouse red blood cell (RBC) suspensions (5%) were incubated with 2%, 3%, and 4% hydrogel extracts at 37 °C for 1 h. PBS and 0.1% Triton X-100 were used as negative and positive controls, respectively. After centrifugation (4 °C, 1000 *g*, 10 min), supernatant absorbance was measured at 540 nm, and hemolysis rate was calculated [61, 62].

### *In vitro* antibacterial activity evaluation

Antimicrobial activity of HA-c-FZ1 hydrogel was evaluated against the Gram-positive bacterium *Staphylococcus aureus* (*S. aureus*) and the Gram-negative bacterium *Escherichia coli* (*E. coli*) using an agar diffusion assay [63]. Bacterial suspensions (100 μL, 1 × 10^5^ CFU/mL) were uniformly spread on Luria-Bertani (LB, Beyotime, China) agar plates. Sterile 6-mm filter paper disks loaded with PBS as the negative control, ampicillin as the positive control, and 2%, 3%, or 4% HA-c-FZ1 hydrogel were aseptically placed onto the inoculated agar surface. Following incubation at 37 °C for 18 h, antimicrobial activity was quantified by measuring the diameter of the inhibition zone (DIZ) [64, 65].

### RNA-seq

All animal procedures were conducted in accordance with the guidelines of the Animal Ethics Committee of Kunming Medical University (Approval No.: kmmu20241591). Full-thickness dorsal wounds with a diameter of 8 mm were generated in C57BL/6 diabetic mice. Wounds were randomly assigned to the control group (PBS) or the HA-c-FZ1 treatment group (*n*  =  4). Skin tissue samples were collected on Day 14 post-intervention. RNA-seq was performed by Majorbio Bio-Pharm Technology Co., Ltd. using the Illumina NovaSeq 6000 platform with paired-end 150 bp sequencing (PE150) [66]. Raw reads were subjected to quality control, aligned to the GRCm38 mouse reference genome using HISAT2, and analyzed for differential gene expression using DESeq2. Differentially expressed genes (DEGs) were identified using thresholds of *P*  <  0.05 and |log_2_FC| > 1. Gene Ontology (GO) and Kyoto Encyclopedia of Genes and Genomes (KEGG) enrichment analyses were performed to identify biological processes and pathways associated with transcriptional changes [67–69].

### Quantitative real-time polymerase chain reaction

RNA-seq results were validated by quantitative real-time polymerase chain reaction (RT-qPCR). From wound margin tissues (three per group), total RNA was extracted with TRIzol reagent (TransGen Biotech, China). cDNA was synthesized via TEasyScript First-Strand cDNA Synthesis SuperMix (TransGen Biotech). Real-time PCR was then run on a LightCycler 480 system (Roche, Switzerland) with PerfectStart Green qPCR SuperMix (TransGen Biotech). Primers were designed for selected DEGs, and their sequences are provided in Table S1. Relative expression was then calculated by the 2^-ΔΔCt^ method with *Gapdh* as the reference gene.

### *In vivo* evaluation of HA-c-FZ1 hydrogel for diabetic wound healing promotion

Six-week-old male C57BL/6 mice (*n*  =  70) were fed a high-fat diet for 8 weeks and then intraperitoneally injected with streptozotocin (STZ, 30 mg/kg/day) for seven consecutive days to induce type 2 diabetes [15, 70]. Mice with stable tail-vein blood glucose concentrations >16.7 mmol/L were randomly allocated, using a computer-generated random sequence, into seven groups (*n*  = 10/group): control (PBS), Tegaderm, 2% HA-c-FZ1, 3% HA-c-FZ1, 4% HA-c-FZ1, HA, and FZ1. Under anesthesia, full-thickness dorsal skin wounds 8 mm in diameter were generated. Each wound was then inoculated with 100 μL of either *S. aureus* or *E. coli* suspension at 1 × 10^6^ CFU/mL, and infection was allowed to establish for 24 h [71]. Infected wounds subsequently received the assigned treatment, and wound closure was photographed and recorded on Days 0, 3, 7, 14, and 21. Quantitative analyses of wound closure, histology, and immunofluorescence were performed by an investigator blinded to treatment allocation.

Wound tissues collected on Days 7 and 14 post-injury were used for enzyme-linked immunosorbent assay (ELISA)-based detection of interleukin-6 (IL-6), tumor necrosis factor alpha (TNF-α), IL-1β, and TGF-β using commercial kits from Neobioscience (China), and were fixed for hematoxylin and eosin (H&E, Solarbio, G1120, China) and Masson’s trichrome staining (Solarbio, G1340, China) [72]. Immunofluorescence staining for arginase (ARG, Affinity, China, DF6657), inducible nitric oxide synthase (iNOS, Affinity, China, AF0199), alpha-smooth muscle actin (α-SMA, Proteintech, China, 14395-1-AP), and vascular endothelial growth factor (VEGF, Proteintech, China, 19003-1-AP) was performed on skin tissue sections collected 14 days post-injury. Stained sections were examined by confocal microscopy (Axio Observer Z1, Zeiss) [53, 71, 73]. Wound blood perfusion was assessed by laser speckle imaging on Days 7 and 14.

### Western blot analysis

Western blotting was performed as described previously [68, 74]. The following primary antibodies were used: nuclear factor kappa B (NF-κB; HUABIO, China, ET1603-12), p-NF-κB (HUABIO, China, ET1604-27), ERK (HUABIO, China, ET1601-29), p-ERK (HUABIO, China, ET1610-13), AKT (HUABIO, China, ET1609-47), p-AKT (HUABIO, China, ET1607-73), FAK (Proteintech, China, 12 636-1-AP), and p-FAK (Proteintech, China, 83 933-1-RR). Protein bands were detected using enhanced chemiluminescence (ECL), quantified with Image Lab software (Bio-Rad), and normalized to GAPDH. For each lane, the normalized abundance of each target protein was calculated by dividing the integrated density of the target band by the integrated density of the corresponding GAPDH band. The resulting normalized values were used for statistical comparisons among groups [75, 76].

### Statistical analysis

All quantitative data are presented as mean ± SD. Statistical significance was determined using one-way or two-way analysis of variance (ANOVA) followed by Tukey’s multiple comparisons test. Statistical analyses were performed using GraphPad Prism (version 10.0, GraphPad Software, USA). *P*  <  0.05 was considered statistically significant.

## Results

### Preparation and characterization of HA-c-FZ1 hydrogel

A multifunctional HA-c-FZ1 hydrogel was designed to improve the local retention, stability, and pathological responsiveness of FZ1 during chronic wound repair. Photo-crosslinked HA served as the structural matrix, with FZ1 incorporated through dynamic Schiff base linkages for pH-regulated peptide release. Rapid gelation was observed upon 365 nm UV exposure of the HA-c-FZ1 precursor solution, showing the feasibility of photo-crosslinking (Figure 1a). HA functionalization was confirmed by ^1^H NMR (Figure 1b), with characteristic resonances for methacrylate double bonds (δ 5.2 to 6.0 ppm) and aldehyde groups (δ 6.5 to 9.2 ppm). Peak-integration analysis showed degree of substitution (DS) of ~16% for methacrylate groups and ~4% for aldehyde groups, confirming controlled modification of HA. SEM imaging showed that higher HA concentrations generated a more continuous interconnected porous network with reduced structural collapse, pointing to improved hydrogel stability (Figure 1c). FTIR spectroscopy supported these results (Figure 1d and Figure S1). Pore size analysis showed larger pores at higher HA concentrations, with 2% HA at 46.2 ± 8.5 μm and 4% HA at 67.1 ± 1.2 μm (Figure 1e). Then a distinct C=O stretching band at ~1712 cm^−1^ confirmed aldehyde formation, whereas conjugating FZ1 to O-HAMA reduced this band and shifted it to ~1715 cm^−1^, matching C=N vibration from imine CH=N bonds. The spectra pointed to aldehyde consumption and reversible Schiff base crosslink formation. These structural improvements supported wound dressing applications, where hydrogels need sufficient stability and mechanical strength during tissue regeneration.

**Figure 1:**
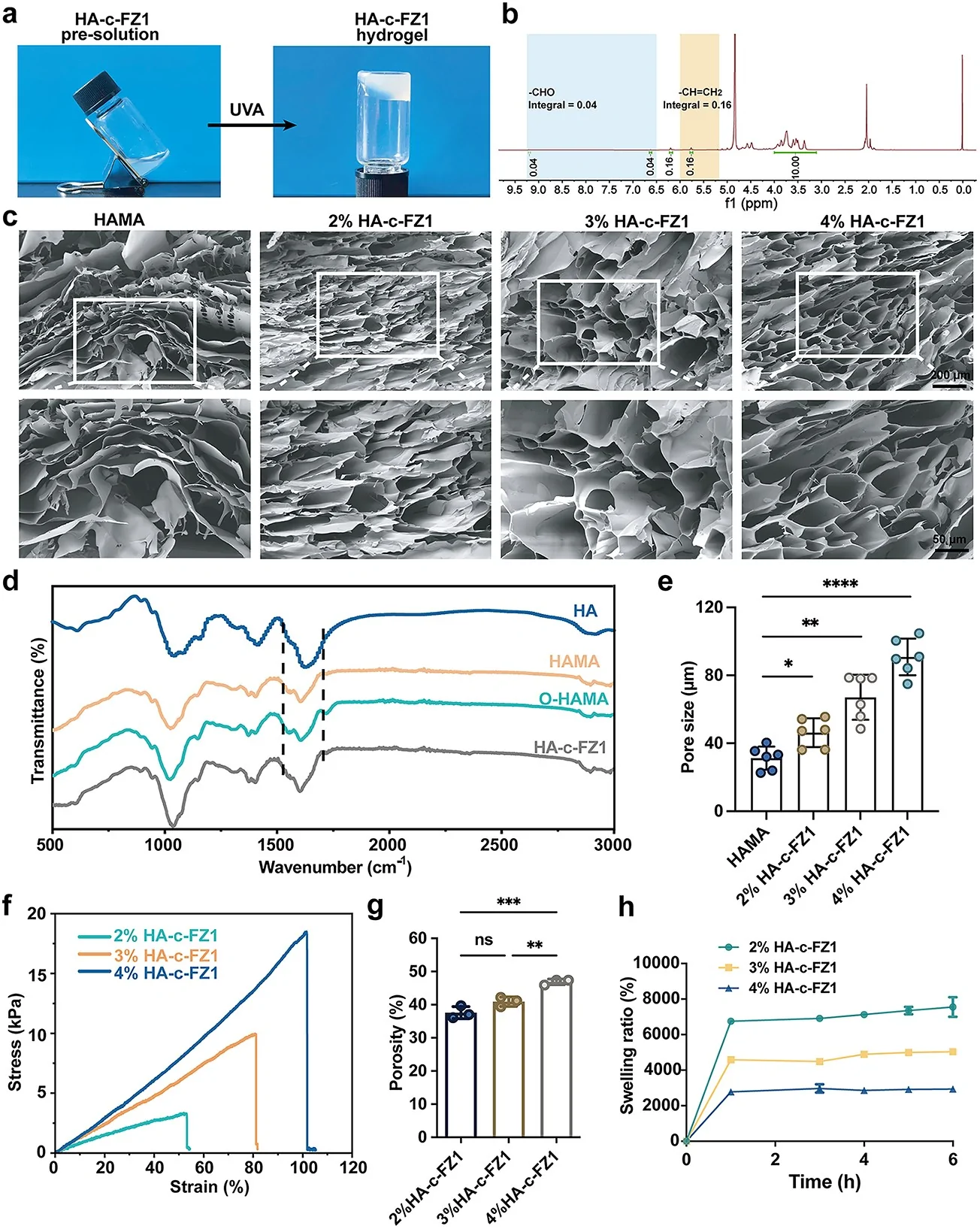
Synthesis and characterization of HA-c-FZ1 hydrogel. (**a**) Schematic representation of HA-c-FZ1 hydrogel formation. The precursor solution (left) underwent rapid gelation upon UVA irradiation, forming a stable hydrogel (right). (**b**) ^1^H NMR (nuclear magnetic resonance) spectra of O-HAMA. Characteristic peaks were assigned to methacrylate vinyl protons (-CH=CH_2_, 5.2–6.0 ppm, integral = 0.16) and aldehyde protons (-CHO, 6.5–9.2 ppm, integral = 0.04). DS was calculated relative to the HA backbone (integral = 1.00, reference), yielding DS = 16% (methacrylate) and 4% (aldehyde). (**c**) SEM images showing cross-sectional morphology of HAMA (methacrylated hyaluronic acid), 2% HA-c-FZ1, 3% HA-c-FZ1, and 4% HA-c-FZ1 hydrogel. Scale bar: 200 μm and 50 μm. (**d**) FTIR spectra of HA, HAMA, O-HAMA, and HA-c-FZ1. (**e–h**) Performance characterization of 2% HA-c-FZ1, 3% HA-c-FZ1, and 4% HA-c-FZ1 hydrogel, including (e) pore size (*n* = 6), (f) stress–strain, (g) porosity (*n* = 3), and (h) swelling ratio (*n* = 3). Data are presented as mean ± SD. Statistical significance was determined using one-way ANOVA followed by Tukey’s multiple comparisons test. ^*^P <  0.05, ^**^P <  0.01, ^***^P < 0.001, ^****^P < 0.001. *Ns* not significant, *HA-c-FZ1* hyaluronic acid conjugated with FZ1 peptide, *UVA* ultraviolet A, *NMR* nuclear magnetic resonance, *O-HAMA* oxidized methacrylated hyaluronic acid, *DS* degree of substitution, *SEM* scanning electron microscopy, *HAMA* methacrylated hyaluronic acid, *FTIR* fourier-transform infrared spectroscopy, *SD* standard deviation, *ANOVA* analysis of variance

FZ1 incorporation into HA-c-FZ1 hydrogel was quantified by determining DLC% and LE% (Figure S2a and b). Among the tested formulations, the 4% HA-c-FZ1 hydrogel exhibited the highest DLC% (12.26% ± 1.39%) and LE% (83.12% ± 3.85%). pH-responsive release behavior was then evaluated for 2%, 3%, and 4% HA-c-FZ1 hydrogel under conditions simulating physiological tissue (pH 7.4), mildly acidic wounds (pH 6.5), and more acidic inflammatory wounds (pH 5.5) (Figure S2c–e). Results demonstrated a significant pH-dependent release profile, with the most rapid and complete release observed at pH 5.5, followed by pH 6.5, and the slowest release observed at pH 7.4. This release pattern indicated that Schiff base crosslinks within the hydrogel network underwent acid-triggered cleavage, thereby accelerating therapeutic peptide liberation under acidic wound conditions. To determine whether pH-responsive release corresponded to the diabetic wound microenvironment, wound pH was monitored throughout healing. During the early inflammatory phase, wound pH remained acidic from Days 1 to 3 (6.28 ± 0.11 to 6.58 ± 0.09), closely matching the pH 6.5 condition used in the release assay (Figure S2f). Although our data on pH and drug release are confined to single time points and consequently fail to delineate the dynamic wound pH changes or the temporal release pattern, the measured pH is consistent with the known inflammatory-phase acidity of diabetic wounds [77]. This consistency supports the use of pH-responsive hydrogels that harness this microenvironmental feature for controlled drug release. As repair progressed, wound pH gradually shifted toward neutrality, reaching 7.08 ± 0.12 by Day 21, consistent with the near-physiological release condition. These findings indicate that HA-c-FZ1 hydrogel release behavior aligns with the pH trajectory of diabetic wound healing and supports pathology-coupled peptide delivery.

The effects of HA molecular weight and oxidation degree on antioxidant activity were further examined. By ABTS^+^ and DPPH assays, HA and O-HAMA with lower molecular weight and less oxidation showed stronger radical-scavenging (Figure S2g–j), a sign that oxidation weakens the intrinsic antioxidant capacity of HA. Other characterization showed that HA-c-FZ1 hydrogel had favorable bioadhesive properties (Figure S2k, l) and robust tensile performance (Figure 1f). Next, hydrogel porosity was found to increase with HA concentration (Figure 1g), matching the enhanced pore-network stability shown by SEM. This likely comes from more polymer chain entanglement with higher concentrations that strengthened the three-dimensional hydrogel network. Increased HA concentration also improved swelling behavior (Figure 1h). The denser crosslinked structure restricted excessive swelling while keeping the hydration capacity needed for wound microenvironment stabilization. pH-regulated release, controlled swelling, antioxidant activity, tissue adhesion, and mechanical stability make HA-c-FZ1 a responsive wound dressing platform that can modulate early inflammation, promote angiogenesis, and support progression to the proliferative phase of repair.

### HA-c-FZ1 hydrogel exhibits excellent biocompatibility

Hemocompatibility and cytocompatibility analyses were performed to evaluate the biosafety of HA-c-FZ1 hydrogel. MTS assays showed that extracts from 2%, 3%, and 4% HA-c-FZ1 hydrogel produced no detectable cytotoxicity in HSFs, HaCaT keratinocytes, RAW264.7 cells, or HUVECs, with relative cell viability remaining above 95% in all tested cell types. The 4% HA-c-FZ1 hydrogel extract also significantly promoted cell proliferation (Figure 2a–d). Hemolysis testing showed low hemolysis rates across all HA-c-FZ1 hydrogel extracts, confirming favorable blood compatibility (Figure 2e). Calcein-AM/PI staining showed >98% viable HaCaT cells after 24 h of exposure to HA-c-FZ1 extracts (Figure 2f), consistent with the MTS results. Histological examination of major organs, including lung, liver, spleen, kidney, and heart, revealed preserved tissue architecture without evident inflammatory infiltration or tissue injury after treatment with 2%, 3%, or 4% HA-c-FZ1 hydrogel extracts (Figure 2g). Cell scratch wound assays further demonstrated that HA-c-FZ1 extracts accelerated cell migration, with higher scratch closure rates at 12 and 24 h than those in the control group (Figure 2h, i). Collectively, these findings establish HA-c-FZ1 as a highly biocompatible multifunctional hydrogel with strong suitability as a bioactive wound dressing for chronic skin wound repair.

**Figure 2:**
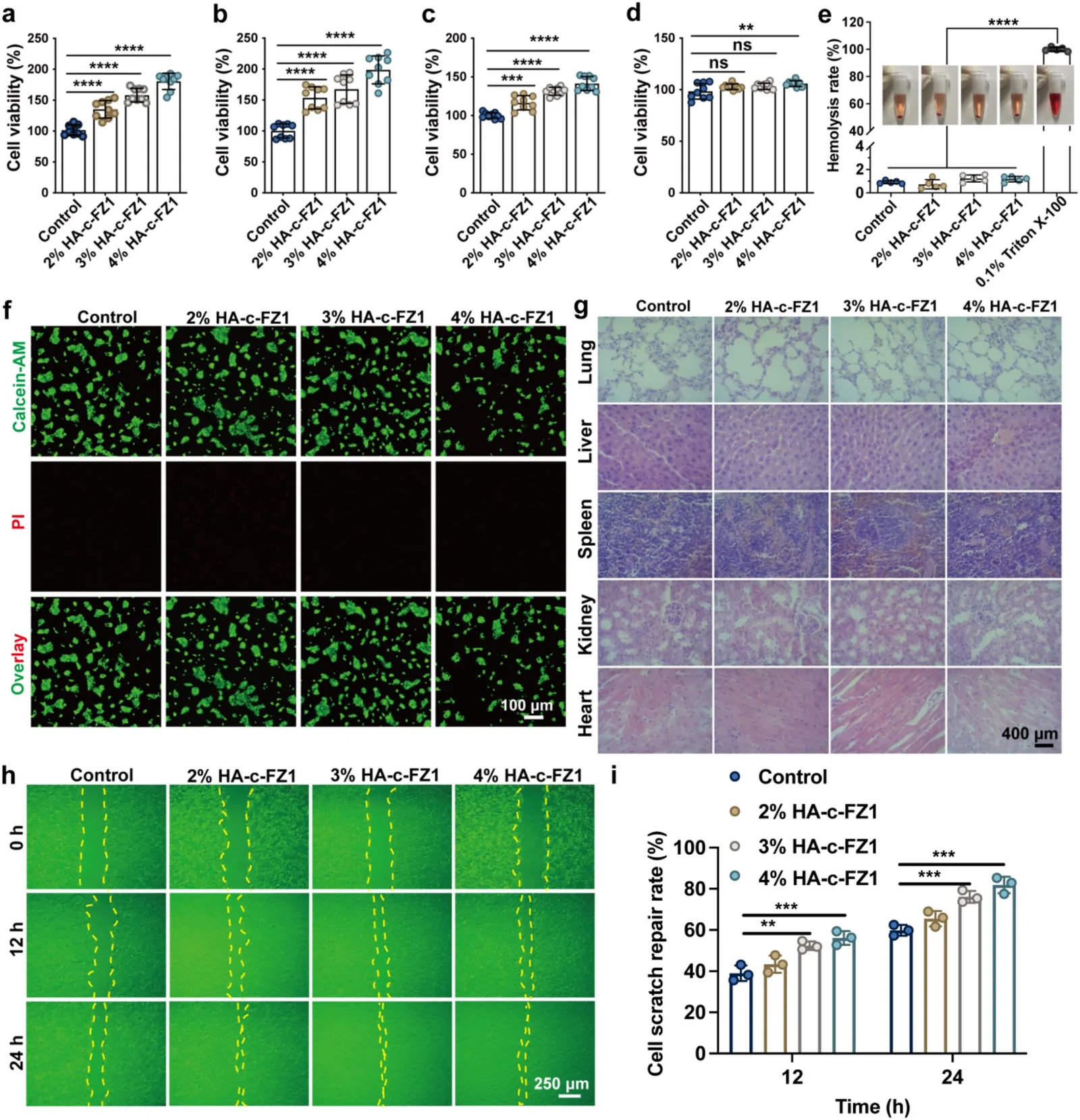
Biocompatibility assessment of HA-c-FZ1 hydrogel. (**a–d**) MTS (3-(4,5-dimethylthiazol-2-yl)-5-(3-carboxymethoxyphenyl)-2-(4-sulfophenyl)-2H-tetrazolium) assays showing the effects of control or 2%, 3%, or 4% HA-c-FZ1 hydrogel extracts on the proliferation of (a) HSFs, (b) HaCaT cells (human keratinocyte cell line), (c) RAW264.7 cells (murine macrophage cell line), and (d) HUVECs, *n* = 9. (**e**) Hemolysis rates in the control group, 2%, 3%, and 4% HA-c-FZ1 hydrogel extract groups, and 0.1% Triton X-100 positive control group, *n* = 5. (**f**) Calcein-AM/PI (calcein acetoxymethyl/propidium iodide) staining of cells treated with control and 2%, 3%, or 4% HA-c-FZ1 hydrogel extracts. Scale bar: 100 μm. (**g**) H&E staining of lung, liver, spleen, kidney, and heart tissues from animals treated with control and 2%, 3%, or 4% HA-c-FZ1 hydrogel extracts. Scale bar: 400 μm. (**h**) Cell scratch assay of keratinocytes cultured with control or 2%, 3%, and 4% HA-c-FZ1 hydrogel extracts. Scale bar: 250 μm. (**i**) Quantitative analysis of keratinocyte scratch closure rate, *n* = 3. Data are presented as mean ± SD. Statistical significance was determined using one-way or two-way ANOVA followed by Tukey’s multiple comparisons test. ^**^P < 0.01, ^***^P <  0.001, ^****^P < 0.0001. *Ns* not significant, *HA-c-FZ1* hyaluronic acid conjugated with FZ1 peptide, *HSFs* human skin fibroblasts, *HaCaT* human keratinocyte cell line, *HUVECs* human umbilical vein endothelial cells, *H&E* hematoxylin and eosin, *SD* standard deviation, *ANOVA* analysis of variance

### *In vitro* antioxidant, anti-inflammatory, and antibacterial activities of HA-c-FZ1 hydrogel

Bacterial colonization, excessive ROS, and nonresolving inflammation together disrupt tissue repair in diabetic wounds [78]. HA-c-FZ1 hydrogel was tested for antibacterial activity against *S. aureus* and *E. coli* by agar diffusion assays. HA-c-FZ1 produced clear inhibition zones against both bacteria, though its activity was lower than ampicillin (Figure 3a). Quantification confirmed this effect (Figure 3b), pointing to intrinsic antibacterial activity. Adding complementary antimicrobial strategies such as photothermal agents could further strengthen infection control in infected wounds. Oxidative stress is another major barrier in diabetic wound repair. Excessive ROS damages cells and disrupts regenerative homeostasis while sustaining NF-κB-driven inflammation. HA is a natural polysaccharide that scavenges radicals, supplying a chemical basis for redox regulation in HA-c-FZ1 hydrogel. In HUVECs exposed to H_2_O_2_, HA-c-FZ1 reduced DCF fluorescence in a concentration-dependent manner, indicating lower intracellular ROS (Figure 3c). ROS levels showed strong suppression across HA-c-FZ1 groups compared with H_2_O_2_ controls (Figure 3d). ABTS^+^ and DPPH assays also confirmed that HA-c-FZ1 extracts have strong radical-scavenging activity (Figure 3e, f), a sign that the platform can manage redox imbalance and break the oxidative stress-inflammation cycle.

**Figure 3:**
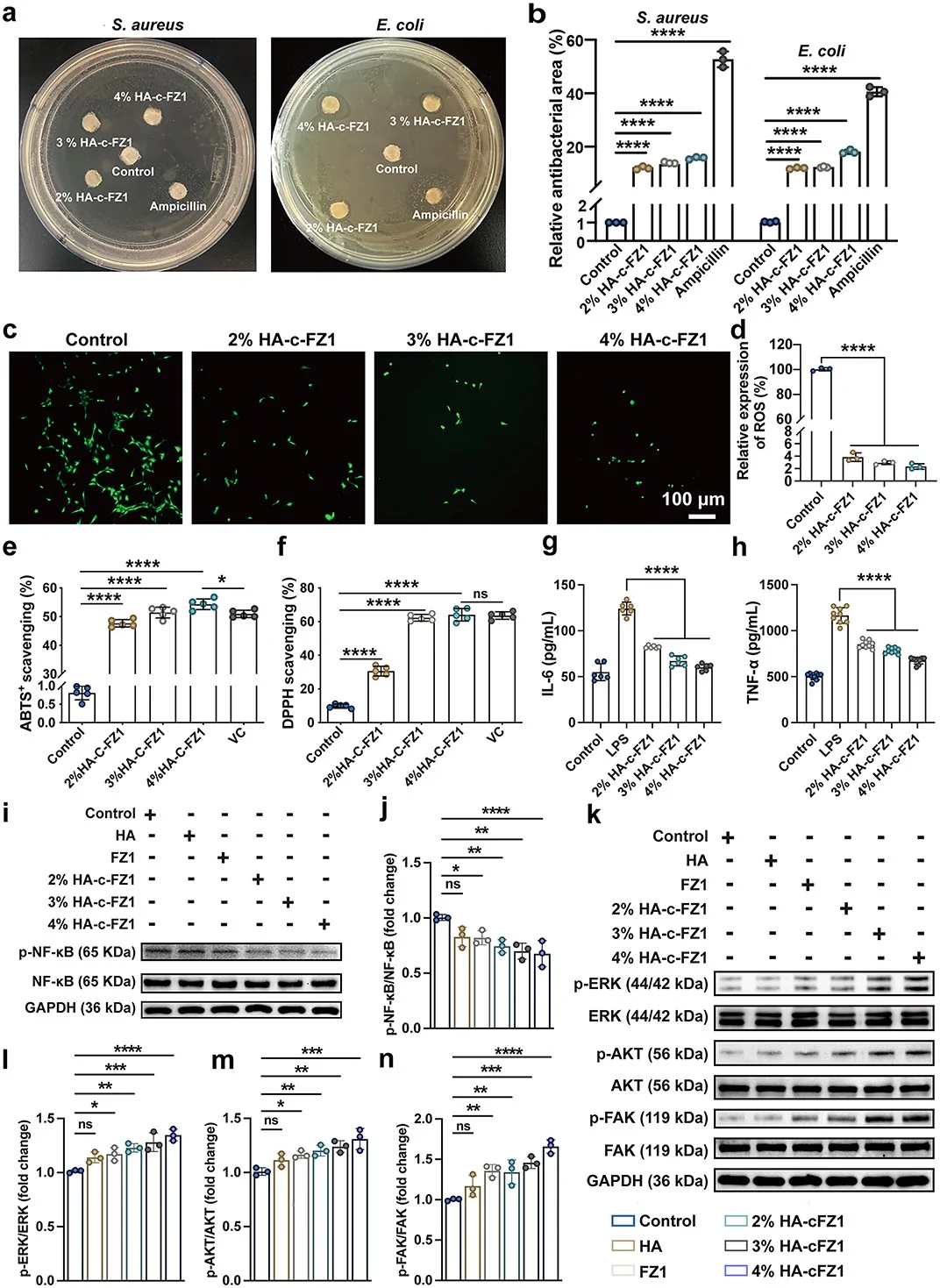
HA-c-FZ1 hydrogel exhibits *in vitro* antioxidant, anti-inflammatory, and antimicrobial activities. (**a**) Antibacterial activity of HA-c-FZ1 hydrogel at different concentrations against *S. aureus* and *E. coli*, with ampicillin used as the positive control. (**b**) Quantification of relative antibacterial area (%) for *S. aureus* and *E. coli* from the assay shown in (a), *n* = 3. (**c**) Intracellular ROS levels in HUVECs treated with HA-c-FZ1 hydrogel extracts, measured using the DCFH-DA (2′,7′-dichlorodihydrofluorescein diacetate) fluorescence probe. Scale bar: 100 μm. (**d**) Quantification of ROS levels, *n* = 3. (**e**, **f**) ABTS^+^ and DPPH radical-scavenging activity (%) of control, 2%, 3%, and 4% HA-c-FZ1 hydrogel extracts, and the vitamin C (VC) positive control, *n* = 5. (**g**, **h**) IL-6 and TNF-α levels (pg/mL) in the supernatants of cells treated with control, LPS (inflammatory inducer), or different hydrogel formulations, *n* = 9. (**i**, **j**) Western blot analysis of the NF-κB (nuclear factor kappa-B) pathway and quantification of p-NF-κB/NF-κB after HA, FZ1, and HA-c-FZ1 treatment (normalized to NF-κB). Data represent three independent experiments (*n* = 3). (**k–n**) Western blot analysis of the MAPK/ERK, AKT, and FAK pathways, with quantification of (l) p-ERK/ERK, (m) p-AKT/AKT, and (n) p-FAK/FAK ratios (each normalized to its corresponding total protein), *n* = 3. Data are presented as mean ± SD. Data are presented as mean ± SD. Statistical significance was determined using one-way ANOVA followed by Tukey’s multiple comparisons test. ^*^P < 0.05, ^**^P < 0.01, ^***^P < 0.001, ^****^P < 0.0001. *Ns* not significant, *HA-c-FZ1* hyaluronic acid conjugated with FZ1 peptide, *ROS* reactive oxygen species, *HUVECs* human umbilical vein endothelial cells, *IL* interleukin, *TNF-α* tumor necrosis factor alpha, *LPS* lipopolysaccharide, *NF-κB* nuclear factor kappa-B, *MAPK* mitogen-activated protein kinase, *ERK* extracellular signal-regulated kinase, *AKT* protein kinase B, *FAK* focal adhesion kinase, *SD* standard deviation, *ANOVA* analysis of variance, *S. aureus Staphylococcus aureus, E. coli Escherichia coli*

Persistent inflammation further compromises cellular function and tissue regeneration in diabetic wounds [79]. Given the antioxidant activity of HA-c-FZ1 hydrogel, its capacity to modulate ROS-mediated inflammatory responses was examined in lipopolysaccharide (LPS)-stimulated RAW264.7 macrophages. ELISA analysis showed that HA-c-FZ1 hydrogel suppressed the secretion of proinflammatory cytokines IL-6 and TNF-α in a concentration-dependent manner, with the strongest inhibition observed in the 4% HA-c-FZ1 group (Figure 3g, h). These findings indicate that HA-c-FZ1 may restore inflammatory balance through potential modulation of macrophage polarization signaling.

Western blotting identified signaling pathways linked to the immunomodulatory and pro-regenerative activity of HA-c-FZ1 (Figure 3i). The 3% and 4% HA-c-FZ1 hydrogel suppressed the NF-κB pathway, reducing the ratio of p-NF-κB to NF-κB compared with controls, a sign that NF-κB activation and inflammatory signaling were suppressed (Figure 3j). Phosphorylation of ERK, AKT, and FAK in HUVECs was examined to determine whether HA-c-FZ1 hydrogel affected proangiogenic signals (Figure 3k). Next, the 4% HA-c-FZ1 hydrogel increased both the p-ERK/ERK, p-AKT/AKT, and p-FAK/FAK ratios (Figure 3l–n), activating the ERK, AKT, and FAK signaling pathways. These results show that HA-c-FZ1 hydrogel supports HUVEC growth and angiogenesis-associated signals with antibacterial, antioxidant, and anti-inflammatory properties *in vitro*, pointing to its potential as a multifunctional platform for chronic wound management.

### *In vivo* evaluation of HA-c-FZ1 hydrogel for diabetic wound healing

The therapeutic efficacy of HA-c-FZ1 hydrogel was evaluated in an STZ-induced type 2 diabetic mouse model with bacterially infected full-thickness skin wounds, following the experimental workflow shown in Figure 4a, b. Serial wound imaging showed that HA-c-FZ1 hydrogel treatment accelerated wound closure over 21 days compared with the control and Tegaderm groups (Figure 4a). The 4% HA-c-FZ1 group showed the most pronounced healing response, with significantly higher wound closure rates than the control group on Day 7 (76.12% ± 1.67% vs. 53.89% ± 5.29%), Day 14 (90.58% ± 2.90% vs. 74.25% ± 4.23%), and Day 21 (96.62% ± 3.68% vs. 85.22% ± 3.75%) (Figure 4c), indicating that HA-c-FZ1 hydrogel markedly accelerated wound repair in infected diabetic skin.

**Figure 4:**
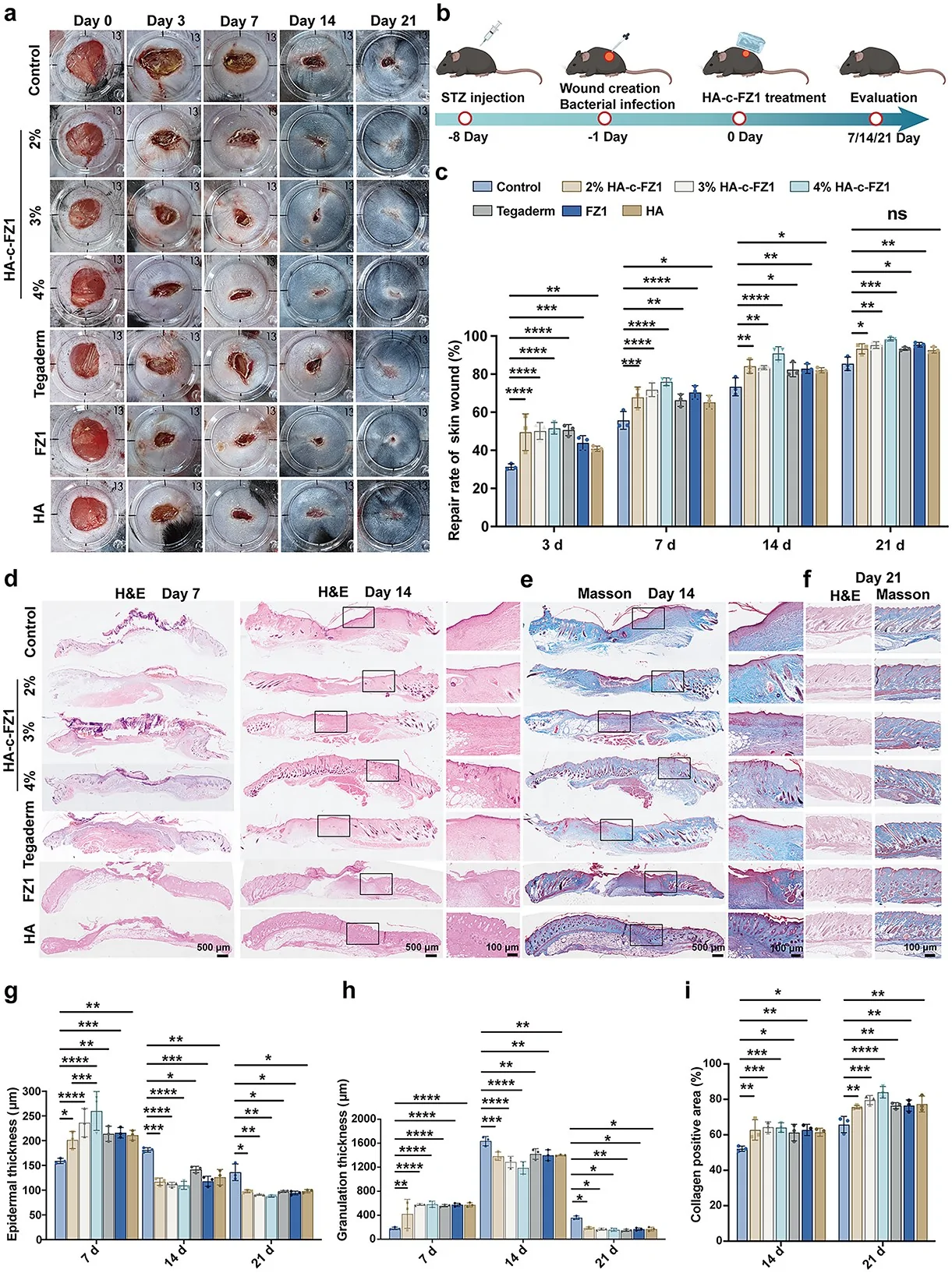
*In vivo* evaluation of HA-c-FZ1 hydrogel for diabetic wound repair in STZ-induced diabetic mice. (**a**) Representative wound images at different time points. (**b**) Experimental timeline. (**c**) Quantification of wound closure, *n* = 3. (d) H&E staining of wound sections on Days 7, 14, and 21. Scale bar: 500 μm. (**e**) Masson’s trichrome staining on Day 14. (**f**) H&E staining and Masson’s trichrome staining of wound sections on Day 21. Scale bars: 500 μm (low-magnification images) and 100 μm (high-magnification images). (**g**, **h**) Quantification of epidermal thickness and granulation tissue thickness on Days 7, 14, and 21, *n* = 6. (**i**) Collagen-positive area fraction on Days 14 and 21, *n* = 3. Data are presented as mean ± SD. Statistical significance was determined using two-way ANOVA followed by Tukey’s multiple comparisons test. ^*^P < 0.05, ^**^P < 0.01, ^***^P < 0.001, ^****^P < 0.0001. *Ns* not significant, *HA-c-FZ1* hyaluronic acid conjugated with FZ1 peptide, *STZ* streptozotocin, *H&E* hematoxylin and eosin, *SD* standard deviation, *ANOVA* analysis of variance

Histological analysis further demonstrated improved tissue reconstruction after HA-c-FZ1 hydrogel treatment (Figure 4d–i). H&E staining showed more advanced re-epithelialization and granulation tissue formation in HA-c-FZ1-treated wounds than in control wounds. On Day 14, epidermal thickness was reduced in the HA-c-FZ1-treated groups, with values of 119.39 ± 7.52 μm for 2% HA-c-FZ1, 73.92 ± 6.42 μm for 3% HA-c-FZ1, and 69.6 ± 6.77 μm for 4% HA-c-FZ1 hydrogel, compared with 182.78 ± 4.54 μm in the control group (Figure 4g). Granulation tissue was also thicker and more organized after HA-c-FZ1 treatment, reaching 835.55 ± 74.66 μm in the 2% HA-c-FZ1 group, 796.66 ± 65.15 μm in the 3% HA-c-FZ1 group, and 769.58 ± 64.39 μm in the 4% HA-c-FZ1 group, compared with 673.99 ± 68.92 μm in the control group (Figure 4h). Masson’s trichrome staining showed a higher collagen-positive area in the 3% and 4% HA-c-FZ1 hydrogel groups on Day 14, suggesting enhanced ECM deposition and remodeling (Figure 4e, f, and  i), both of which were important for wound healing and tissue regeneration. By Day 21, wound closure rates had largely converged across all treatment groups, with significant differences maintained primarily relative to the control group. This temporal pattern indicated that HA-c-FZ1, particularly the 3% and 4% formulations, accelerated infected diabetic wound repair by advancing the transition from inflammation to tissue formation and remodeling, with improved re-epithelialization, granulation tissue organization, and collagen deposition (Figure 4f–i).

### Transcriptomic analysis reveals mechanisms underlying HA-c-FZ1 hydrogel-mediated wound repair

Transcriptomic analysis was performed on skin tissues collected from HA-c-FZ1-treated and control diabetic mice on Day 14 after model establishment to define the molecular programs associated with accelerated wound repair. Principal component analysis (PCA) revealed distinct separation between the two groups along PC1, which explained 54.61% of total variance (Figure 5a), indicating a marked transcriptional shift after HA-c-FZ1 treatment. Differential expression analysis identified 262 upregulated and 735 downregulated DEGs in the HA-c-FZ1 group relative to the control group (Figure 5b). The volcano plot and hierarchical heatmap further illustrated the broad remodeling of gene expression patterns induced by HA-c-FZ1 hydrogel (Figure 5c, d). GO enrichment analysis indicated that DEGs were mainly involved in immune-related biological processes, including “immune system process”, “regulation of immune system process”, and “defense response” (Figure 5e), suggesting that HA-c-FZ1 hydrogel modulates the wound immune microenvironment during tissue repair. KEGG pathway analysis further identified enrichment of inflammation- and immunity-associated pathways, including “cytokine-cytokine receptor interaction”, “NOD-like receptor signaling pathway”, and “TNF signaling pathway” (Figure 5f), which are closely linked to inflammatory regulation, host defense, and tissue remodeling. In parallel, genes upregulated after HA-c-FZ1 hydrogel treatment ( e.g. *Wnt10b*, *Ccna2*, *Tfrc*, *Ccne2*, *Kitl*) were enriched in the mitogen-activated protein kinase (MAPK) and PI3K-AKT signaling pathways, both of which are strongly associated with cellular proliferation, migration, and regenerative repair ( Figure S3a, b). This transcriptional pattern is consistent with *in vitro* evidence showing HA-c-FZ1-mediated activation of ERK, AKT, and FAK signaling (Figure 3i–n). RT-qPCR validation of core inflammation-related DEGs within the enriched pathways, including *Tnf*, *Il1b*, and *Cxcl2*, confirmed expression trends consistent with the RNA-seq data and demonstrated significant downregulation of these proinflammatory genes after HA-c-FZ1 treatment (Figure S4a–l). Together with the *in vivo* wound-repair outcomes, these transcriptomic findings indicate that HA-c-FZ1 hydrogel promotes diabetic wound healing by orchestrating immune regulation, inflammatory resolution, ECM remodeling, cell proliferation, and migration, and anti-infective responses through defined molecular pathways (Figure 5g).

**Figure 5:**
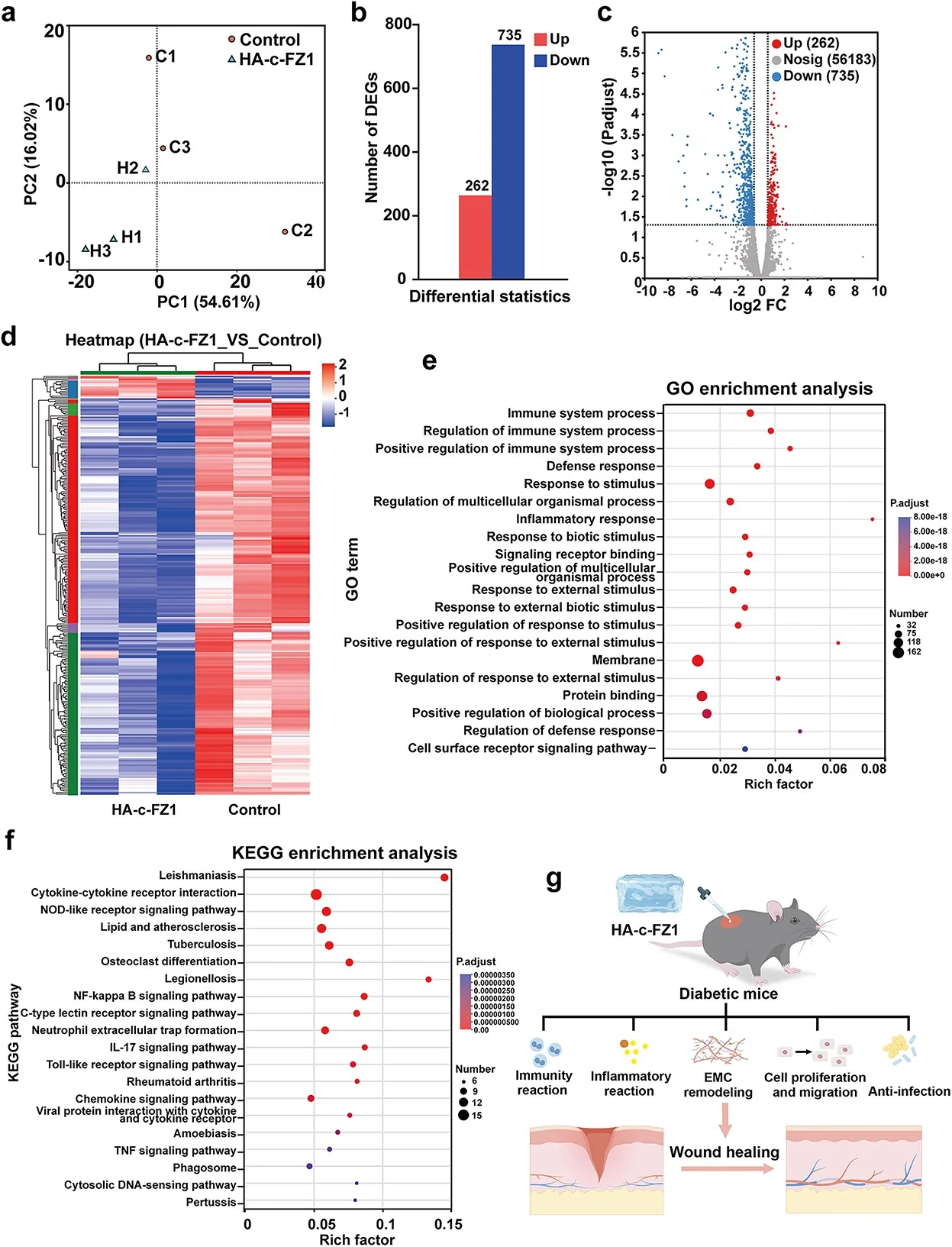
Transcriptomic analysis and proposed mechanism of HA-c-FZ1 hydrogel-mediated diabetic wound healing. (**a**) PCA of gene expression profiles from control and HA-c-FZ1-treated groups, *n* = 3. (**b**) Statistical summary of DEGs between control and HA-c-FZ1-treated groups. (**c**) Volcano plot showing DEG distribution. (**d**) Heatmap showing hierarchical clustering of DEGs between HA-c-FZ1-treated and control groups. (**e**) GO enrichment analysis of DEGs. (**f**) KEGG pathway enrichment analysis of DEGs. (**g**) Schematic illustration of the proposed mechanism by which the HA-c-FZ1 hydrogel promotes diabetic wound repair. Data are from three independent experiments. *HA-c-FZ1* hyaluronic acid conjugated with FZ1 peptide, *PCA* principal component analysis, *DEGs* differentially expressed genes, *GO* gene ontology, *KEGG* kyoto encyclopedia of genes and genomes

### HA-c-FZ1 suppresses inflammation by regulating macrophage polarization and inhibiting NF-κB signaling

HA-c-FZ1 hydrogel was tested for immunomodulatory and antibacterial effects to elucidate how they reshape the infected diabetic wound microenvironment. Western blotting showed that HA-c-FZ1 hydrogel reduced the ratio of p-NF-κB to NF-κB in wound tissue (Figure 6a, b), a sign that NF-κB activation was suppressed and inflammatory cytokine production reduced. ELISA analysis showed that HA-c-FZ1 hydrogel, especially the 3% and 4% formulations, lowered proinflammatory cytokines including IL-1β, IL-6, and TNF-α and raised the anti-inflammatory cytokine TGF-β (Figure 6c–f). These changes pointed to an immunomodulatory shift from persistent inflammation to a reparative state.

**Figure 6:**
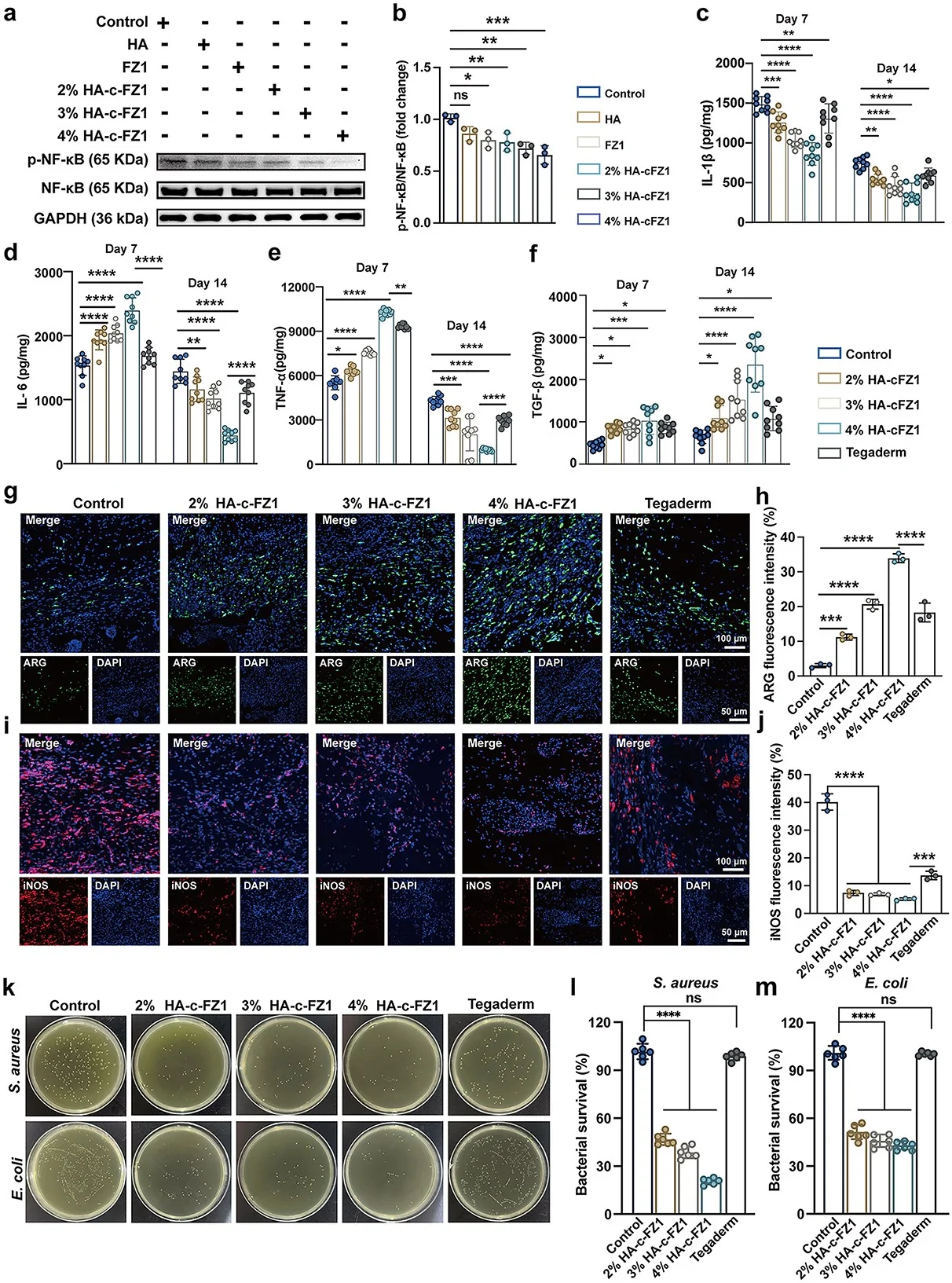
Immunomodulatory and antibacterial effects of HA-c-FZ1 hydrogel during diabetic wound repair. (**a, b**) Western blot analysis (a) and quantification (b) of p-NF-κB and NF-κB in wound tissues on Day 14, *n* = 3. (**c–f**) Levels of IL-1β (c), IL-6 (d), TNF-α (e), and TGF-β (transforming growth factor beta) (f) in wound tissues on Days 7 and 14, *n* = 9. (**g–j**) Immunofluorescence staining and quantification of ARG (g and h) and iNOS (i and j) in wound tissues on Day 14. Merged images show DAPI-labeled nuclei (blue), ARG (green), and iNOS (red). Fluorescence intensity was quantified as a percentage (%). Scale bars: 100 μm (merged) and 50 μm (single channels), *n* = 3. (**k–m**) Antibacterial activity of HA-c-FZ1 hydrogel, including representative agar plates (k) and quantification of bacterial survival (%) for *S. aureus* (l) and *E. coli* (m), *n* = 9. Data are presented as mean ± SD. Statistical significance was determined using one-way or two-way ANOVA followed by Tukey’s multiple comparisons test. ^*^P < 0.05, ^**^P < 0.01, ^***^P < 0.001, ^****^P < 0.0001. *Ns* not significant, *HA-c-FZ1* hyaluronic acid conjugated with FZ1 peptide, *NF-κB* nuclear factor kappa-B, *IL* interleukin, *TNF-α* tumor necrosis factor alpha, *TGF-β* transforming growth factor beta, *ARG* arginase, *iNOS* inducible nitric oxide synthase, *DAPI* 4',6-diamidino-2-phenylindole, *SD* standard deviation, *ANOVA* analysis of variance, *S. aureus Staphylococcus aureus, E. coli Escherichia coli*

Immunofluorescence staining confirmed this immunoregulatory effect. HA-c-FZ1 increased ARG expression and lowered iNOS expression in wound tissues, a sign that macrophages shifted from a proinflammatory M1 toward a reparative M2 phenotype (Figure 6g–j). Antibacterial assays showed that HA-c-FZ1 inhibited *S. aureus* and *E. coli*, among which the 3% and 4% hydrogels had the strongest activity (Figure 6k–m). These results show that HA-c-FZ1 promotes diabetic wound healing through NF-κB pathway suppression, shifting macrophage polarization toward a reparative phenotype while reducing bacterial burden. These changes create a microenvironment more permissive for tissue regeneration.

### HA-c-FZ1 improves wound perfusion and promotes angiogenesis

Angiogenic signaling and wound perfusion were assessed to see how these hydrogels support tissue regeneration in diabetic wounds. Western blotting of wound tissues at Day 14 showed that HA-c-FZ1 hydrogel activated the ERK, AKT, and FAK signaling pathway, with the strongest responses in the 3% and 4% groups (Figure 7a–d). Phosphorylation ratios of ERK, AKT, and FAK were higher than in controls, a sign that HA-c-FZ1 hydrogel regulates these pathways to support cell proliferation and migration. Immunofluorescence staining showed increased and organized α-SMA^+^ myofibroblast cells in wounds treated with HA-c-FZ1 hydrogel, a sign of enhanced matrix remodeling and wound contraction (Figure 7e, f). VEGF fluorescence intensity increased after HA-c-FZ1 treatment, especially in the 3% and 4% groups (see Figure 7g, h), a sign of improved pro-angiogenic activity within the wound bed. Laser Doppler imaging showed that blood perfusion increased on Days 7 and 14, with higher signals in the 3% and 4% groups than in controls (Figure 7i, j). These data show that HA-c-FZ1 hydrogel improve wound healing by activating the ERK, AKT, and FAK signaling pathways, supporting myofibroblast formation, increasing VEGF expression, and boosting blood perfusion. Together, these effects create a regenerative microenvironment that supports diabetic wound healing.

**Figure 7:**
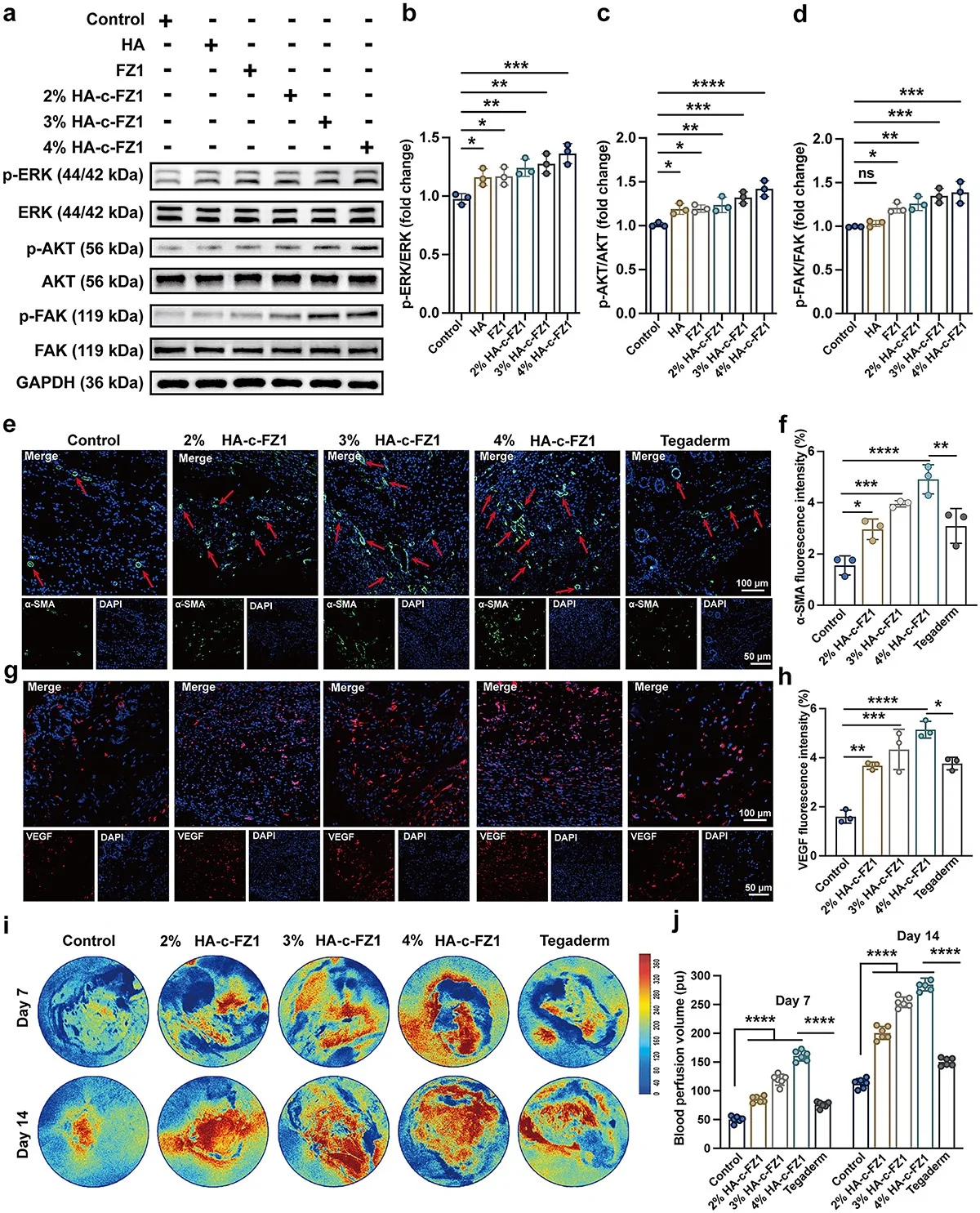
HA-c-FZ1 hydrogel regulates pro-regenerative signaling pathways and promote angiogenesis during wound repair. (**a**) Western blot analysis of p-ERK, ERK, p-AKT, AKT, p-FAK, and FAK in wound tissues collected on Day 14 from mice treated with control, HA, FZ1, 2% HA-c-FZ1 hydrogel, 3% HA-c-FZ1 hydrogel, or 4% HA-c-FZ1 hydrogel. (**b–d**) Quantification of p-ERK/ERK (b), p-AKT/AKT (c), and p-FAK/FAK (d) ratios from the western blot analysis shown in (a), *n* = 3. (**e**, **f**) Immunofluorescence staining of α-SMA (alpha-smooth muscle actin) in wound tissues on Day 14 and quantification of α-SMA fluorescence intensity (%). Green indicates α-SMA staining, red arrows indicate positive signal, and blue indicates DAPI-labeled nuclei, *n* = 3. Scale bars: 100 μm (merged) and 50 μm (single channel), *n* = 3. (**g**, **h**) Immunofluorescence staining of VEGF in wound tissues on Day 14 and quantification of VEGF fluorescence intensity (%), *n* = 3. (**i**, **j**) Laser Doppler blood perfusion imaging of wound tissues on Days 7 and 14 (i) and quantification of blood perfusion volume (j), *n* = 6. Scale bars: 100 μm (merged) and 50 μm (single channel). Data are presented as mean ± SD. Statistical significance was determined using one-way or two-way ANOVA followed by Tukey’s multiple comparisons test. ^*^P < 0.05, ^**^P < 0.01, ^***^P < 0.001, ^****^P < 0.0001. *Ns* not significant, *HA-c-FZ1* hyaluronic acid conjugated with FZ1 peptide, *ERK* extracellular signal-regulated kinase, *AKT* protein kinase B, *FAK* focal adhesion kinase, *α‑SMA* alpha-smooth muscle actin, *DAPI* 4',6-diamidino-2-phenylindole, *VEGF* vascular endothelial growth factor, *SD* standard deviation, *ANOVA* analysis of variance

## Discussion

Hyperglycemia-associated mitochondrial dysfunction drives a pathological cycle in DFUs that raises oxidative damage and prolongs NF-κB activation [80]. Microvascular injury reduces perfusion, creating hypoxic niches that favor anaerobic infection, slow keratinocyte migration, and impair tissue repair [81]. This combined pathology explains why single-axis treatments have limited efficacy. Bioactive peptides, including amphibian-derived ones, offer multi-target potential, but clinical use remains limited by poor retention, rapid degradation, and weak delivery control. Peptide-loaded hydrogel systems are promising platforms for chronic wound treatment [82]. In this study, FZ1 was integrated into a photocrosslinkable HA hydrogel using a pH-responsive Schiff base design, which provides local peptide retention and acid-triggered release with comprehensive control of the DFU microenvironment. The approach couples peptide delivery to pathological wound cues with antioxidant, antimicrobial, anti-inflammatory, and pro-angiogenic functions in a single adaptive dressing. Unlike conventional peptide-based hydrogels, HA-c-FZ1 hydrogel showed stronger microenvironment responsiveness with sustained local FZ1 availability, which may preserve therapeutic effects throughout the extended and highly unpredictable repair process typical of DFUs.

The HA-c-FZ1 hydrogel was made by coupling FZ1 to photocrosslinkable HA through dynamic Schiff base linkages for pH-sensitive release of peptide under acidic diabetic wound conditions. The biphasic release profile reflected FZ1 in two states within the matrix; the early phase came from physically entrapped or weakly associated peptide, and the sustained phase involved FZ1 conjugated through acid-labile Schiff base linkages. Release experiments at pH 5.5, 6.5, and 7.4 supported this mechanism, in which acidic conditions accelerated peptide liberation through pH-triggered Schiff base hydrolysis, with release behavior that matched the acidic wound microenvironment. The photocrosslinkable HA matrix also allowed fast *in situ* gelation and conformal wound coverage, and the porous hydrogel architecture allowed cellular infiltration and exudate management. Beyond structure, HA-c-FZ1 hydrogel remodeled the wound microenvironment by scavenging ROS, lowering oxidative stress while blocking macrophage inflammation through the NF-κB pathway. Lyophilized HA-c-FZ1 hydrogel retained their properties and bioactivity for ≥ 6 months at −20 °C; reconstituted hydrogels remained stable for 7 days at 4 °C, with no notable alterations in swelling, mechanical performance, or release kinetics. Favorable biocompatibility and support of keratinocyte migration, together with these intrinsic multifunctional properties, reconditioned the diabetic wound bed. This provided a mechanistic basis for the synergistic healing effects seen with pH-triggered FZ1 release.

In STZ-induced diabetic mice, HA-c-FZ1 hydrogel promoted wound closure. By Day 14, the 4% hydrogel had achieved 90.58% ± 2.90% closure. Enhanced re-epithelialization, more organized granulation tissue, and greater collagen deposition were seen on histological analysis. Thicker epidermis and more organized granulation tissue at higher HA concentrations show better mechanical support and ECM remodeling. These outcomes reflect reprogramming of the wound bed at the molecular scale. Transcriptomic analysis revealed downregulation of key proinflammatory genes, including *Tnf* and *Il1b*, in enriched pathways such as “cytokine-cytokine receptor interaction” and “TNF signaling pathway”, which provide a molecular basis for NF-κB pathway suppression and reduced inflammatory cytokine production. HA-c-FZ1 hydrogel also drove macrophage polarization toward a reparative M2 phenotype, with higher ARG and lower iNOS expression. This immunological shift limited chronic inflammation, promoted tissue remodeling, and increased TGF-β while reducing IL-1β, IL-6, and TNF-α.

Defective angiogenesis blocks diabetic wound repair because insufficient vascular regeneration limits oxygen and nutrient delivery to the wound bed [83, 84]. HA-c-FZ1-treated wounds showed upregulated genes in the MAPK and PI3K-AKT pathways, including *Wnt10b*, *Ccna2*, *Tfrc*, *Ccne2*, and *Kitl*, linked to FAK-AKT-ERK activation, a network that supports cell survival, migration, and angiogenesis. This activation improved vascular and stromal remodeling, with more α-SMA^+^ myofibroblast-like cells, higher VEGF expression, and better blood perfusion by laser Doppler imaging. These effects indicate that HA-c-FZ1 hydrogel suppresses inflammation, restores pro-angiogenic signaling and local perfusion, and addresses the vascular insufficiency that drives chronic diabetic wound persistence.

The therapeutic efficacy of HA-c-FZ1 hydrogel appears to arise from the synergistic interaction between the HA hydrogel matrix and pH-responsive FZ1 delivery. The photo-crosslinked HA network provides immediate wound coverage, mechanical support, hydration control, and a permissive scaffold for cell migration. In parallel, acid-triggered FZ1 release increases local peptide availability within the inflammatory diabetic wound site, where pro-regenerative signaling is most needed. This temporally regulated delivery works together with the intrinsic anti-inflammatory and ROS-scavenging properties of the HA scaffold to suppress NF-κB signaling and shift macrophage polarization toward a reparative M2 phenotype. Concurrent activation of the FAK-AKT-ERK axis further promotes angiogenesis and vascular restoration. Thus, HA and FZ1 do not function as separate therapeutic elements; instead, they form an integrated system that couples immune microenvironment remodeling with vascular reconstruction, thereby disrupting the self-perpetuating cycle of inflammation, oxidative stress, poor perfusion, and defective repair.

Despite these promising results, several limitations should be considered. First, the exclusive use of male mice, while controlling for hormonal variability, limits extrapolation to female populations and does not fully capture the pathophysiological, anatomical, and microbiological heterogeneity of human DFUs. Second, the moderate intrinsic antibacterial activity of HA-c-FZ1 hydrogel observed *in vitro*, which remained lower than that of ampicillin, represents an important translational limitation. Chronic wounds, particularly DFUs, frequently contain polymicrobial communities and antimicrobial-resistant pathogens; therefore, a moderately active antibacterial agent may fail to achieve adequate bioburden control in clinical settings, increasing the risk of persistent infection, delayed repair, or treatment failure. Nevertheless, the sustained-release behavior of HA-c-FZ1 hydrogel and its favorable modulation of the wound microenvironment, including ROS scavenging, inflammatory attenuation, and angiogenic stimulation, may partially compensate for this limitation by maintaining local therapeutic activity and supporting host-mediated bacterial clearance. Future designs could strengthen antibacterial performance through integration of complementary strategies, including photothermal agents, broad-spectrum antimicrobial peptides, antimicrobial metal ions (e.g. Ag^+^ or Zn^2+^), or conventional antibiotics to enhance activity against multidrug-resistant organisms. Third, the absence of long-term follow-up beyond 21 days and validation in large-animal wound models restricts assessment of durable therapeutic efficacy, scar remodeling, recurrence risk, and clinical translatability. Addressing these issues will be essential for defining the translational value of HA-c-FZ1 hydrogel in chronic wound management.

## Conclusions

HA-c-FZ1 hydrogel is a pH-responsive hydrogel dressing for diabetic wound repair that coordinates peptide delivery with microenvironmental regulation. It combines acid-triggered FZ1 release with a photo-crosslinked HA scaffold to promote antibacterial activity, reduce oxidative stress, suppress NF-κB-associated inflammation, redirect macrophage polarization toward a healing M2 phenotype, and enhance FAK-AKT-mediated angiogenic signaling. This design accelerates tissue repair through immune remodeling and vascular regeneration, making it a promising platform for diabetic wound healing.

## Ethics approval and consent to participate

All animal procedures were conducted in accordance with the guidelines of the Animal Ethics Committee of Kunming Medical University (Approval No.: kmmu20241591).

## Supplementary Material

Supplementary_file_tkag046

Supplementary_Data_1_tkag046
