# Supplementary material for: Multifunctional pH-responsive HA-c-FZ1 hydrogel presents a promising therapeutic strategy for diabetic skin wounds
Source: Burns Trauma. 2026 Jul 5;14:tkag046. doi: 10.1093/burnst/tkag046 (PMC13630460; doi:10.1093/burnst/tkag046)
Supplement: Supplementary file 1 [file supplementary_file_tkag046.pdf]

Supporting Information

Table S1

Gene names	Forward primer Sequence (5'->3')	Reverse primer Sequence (5'->3')
Cd14	GAAGCAGATCTGGGGCAGTT	CGCAGGGCTCCGAATAGAAT
Gadd45b	TGTGCATAAGTCAGCGGAGG	ATGTGCTGTAGCTGCGAAGT
Tnf	GATCGGTCCCCAAAGGGATG	CCACTTGGTGGTTTGTGAGTG
Il1b	TGCCACCTTTTGACAGTGATG	TGATGTGCTGCTGCGAGATT
Nfkbia	CCTGACCTGGTTTCGCTCTT	AGGGGGAGTAGCCTTGGTAG
Cxcl3	GAAAGGAGGAAGCCCCTCAC	ACACATCCAGACACCGTTGG
Ptags2	CATCCCCTTCCTGCGAAGTT	GGCCCTGGTGTAGTAGGAGA
Ccl4	CCCAGCTCTGTGCAAACCTA	CCATTGGTGCTGAGAACCCT
Cxcl2	GCTGTCCCTCAACGGAAGAA	CAGGTACGATCCAGGCTTCC
GAPDH	CCCTTAAGAGGGATGCTGCC	TACGGCCAAATCCGTTCACA

Table S1. Primer sequences used for quantitative real-time PCR (RT-qPCR). GAPDH served as the internal control.

Fig. S1

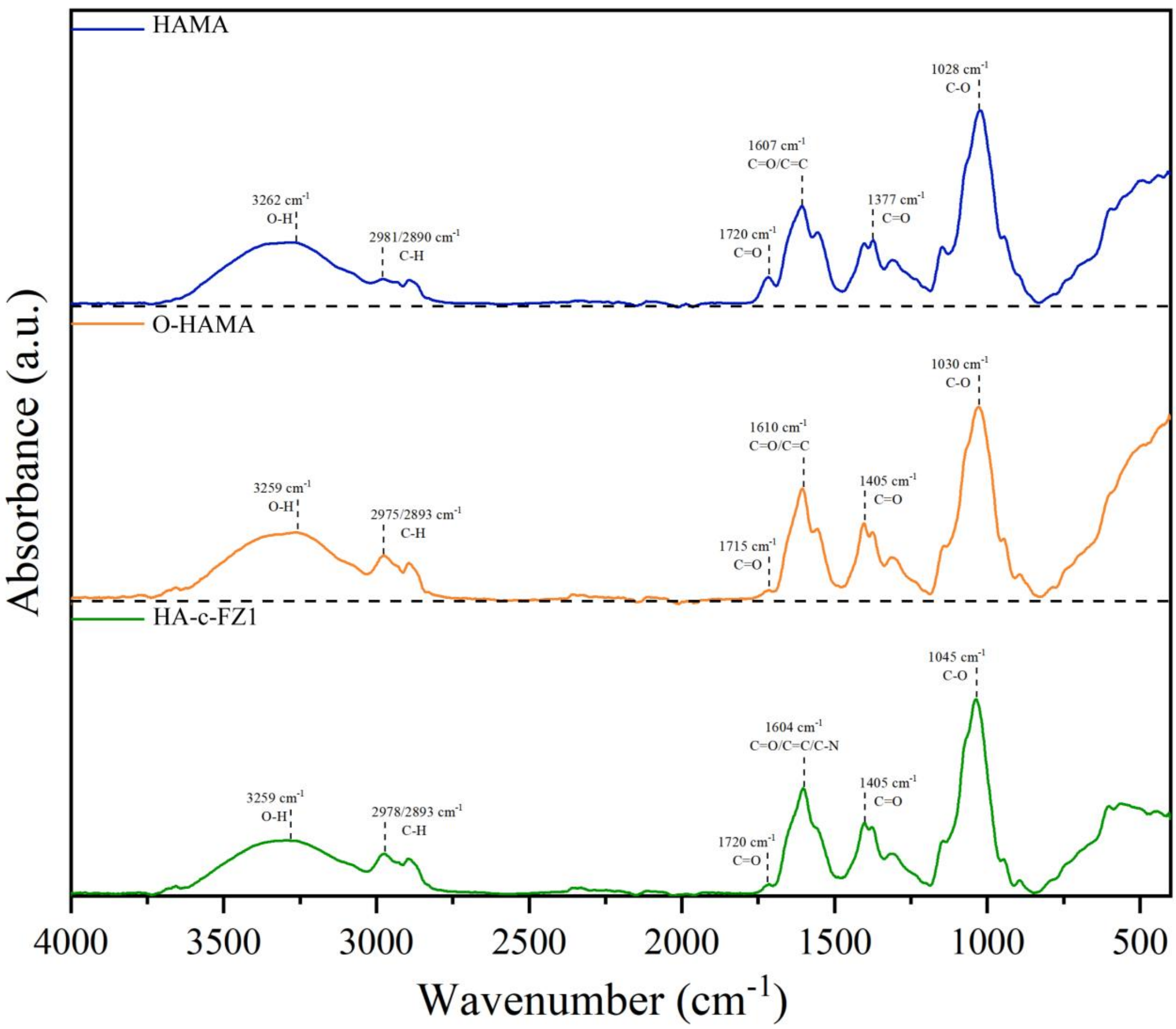

Fig. S1. Presenting the Fourier transform infrared (FTIR) spectra of HAMA (blue line), O-HAMA (orange line), and the final conjugate HA-c-FZ1 (green line) over the range 4000–500 cm^{-1}. Characteristic bands are visible: a broad absorption around 3200–3400 cm^{-1} (O–H/N–H stretch), the C–H stretch near 2900 cm^{-1}, and the ester C=O stretch at approximately 1720 cm^{-1} in HAMA. The spectrum of O-HAMA shows a clear shift and broadening in the 1720 cm^{-1} region, consistent with the introduction of aldehyde C=O groups. Further changes are observed in the amide I (around 1650 cm^{-1}) and amide II (near 1550 cm^{-1}) regions after reaction with the FZ1 peptide, confirming the formation of the HA-c-FZ1 conjugate via a Schiff base linkage.

Fig. S2

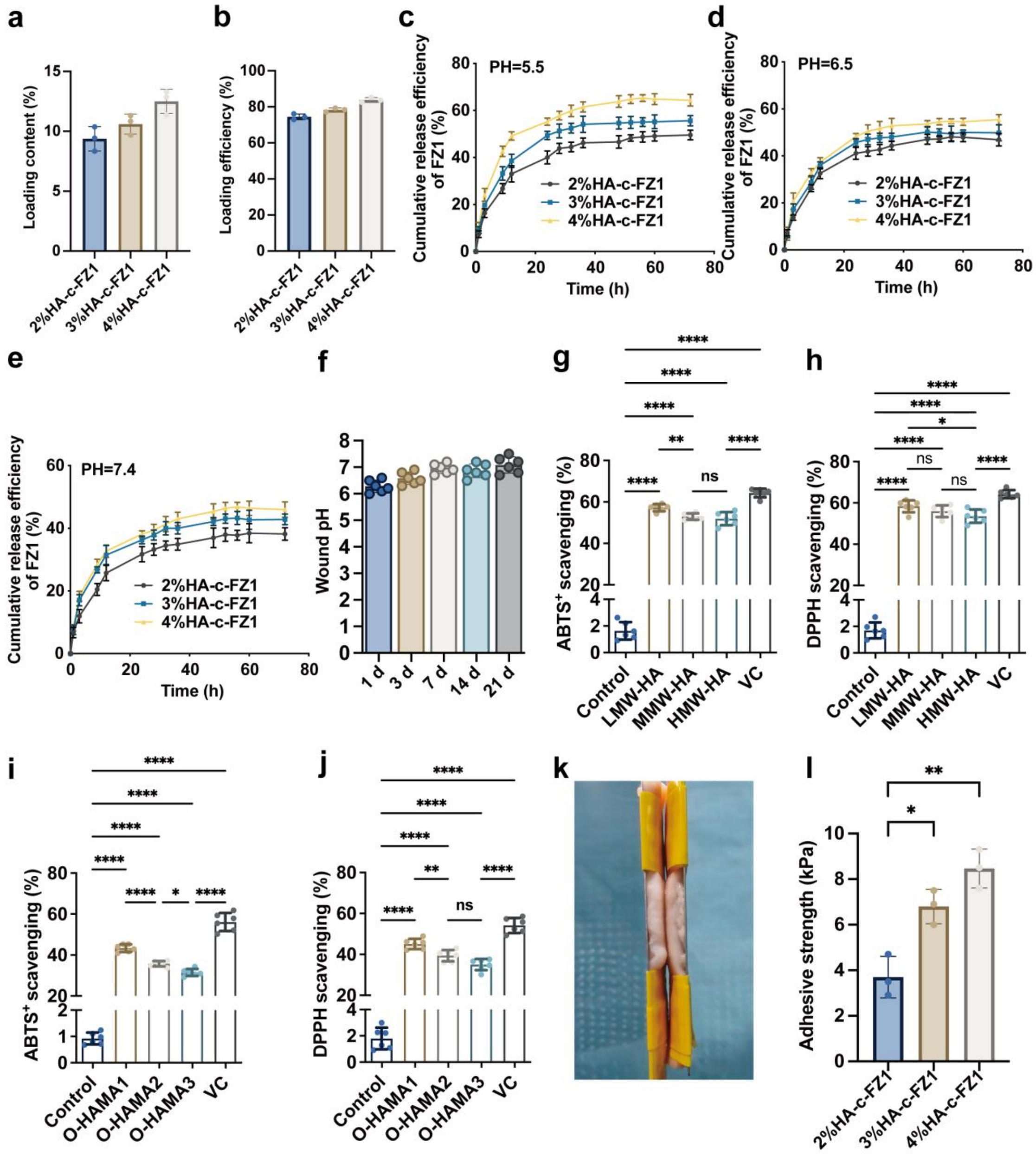

Fig. S2. Material characterization and functional assessments of HA-c-FZ1 hydrogels. a-b. Determination of peptide FZ1 loading content and loading efficiency at different HA concentrations, n=3. **c-e.** pH-responsive release of FZ1 from HA-c-FZ1 (2%, 3%, and 4%) under different pH conditions (5.5, 6.5, and 7.4), n=6. **f.** Wound pH dynamics in STZ-induced diabetic mice. Wound surface pH was measured at Days 1, 3, 7, 14, and 21 post-wounding (n=6). **g-h.** $ABTS^+$ and DPPH assays evaluating the antioxidant capacity of HA with different molecular weights, n=6. **i-j.** $ABTS^+$ and DPPH assays evaluating the antioxidant capacity of O-HAMA with

varying degrees of oxidation, n=6. **k-l.** Bioadhesion testing of HA-c-FZ1 (2%, 3%, and 4%), n=3. Data are expressed as mean ± SD. Statistical significance was determined by two-tailed Student's t test or one-way ANOVA. **$p < 0.01$, ***$p < 0.001$, ****$p < 0.0001$.

Fig. S3

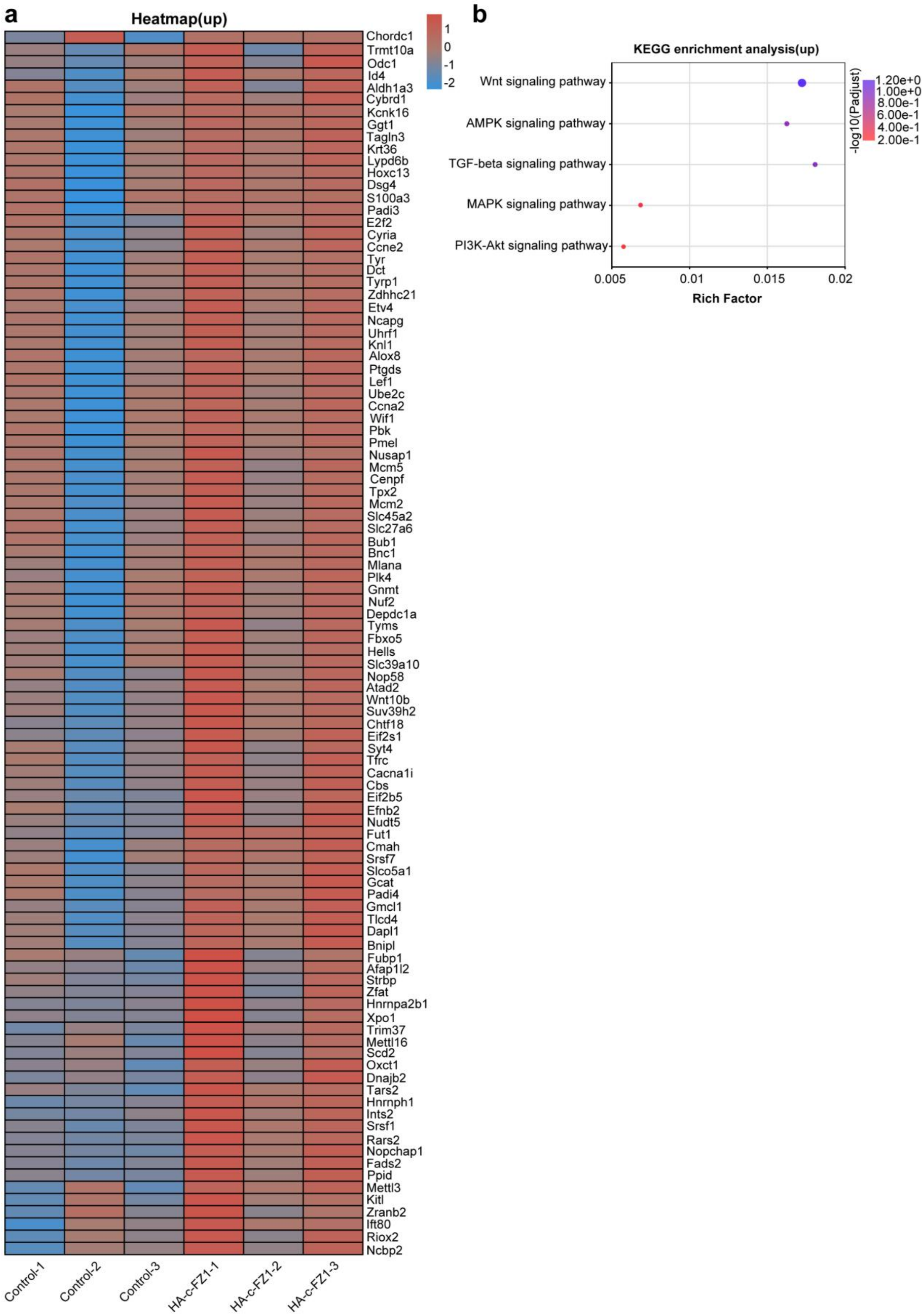

Fig. S3. Upregulated genes and related pathway analysis after HA-c-FZ1 treatment. a. Heatmap displaying upregulated gene expression in HA-c-FZ1 vs. control, with red/blue indicating higher/lower expression. **b.** KEGG enrichment plot showing key pathways (Wnt,

AMPK, TGF-β, MAPK, PI3K-Akt) modulated by the upregulated genes.

Fig. S4

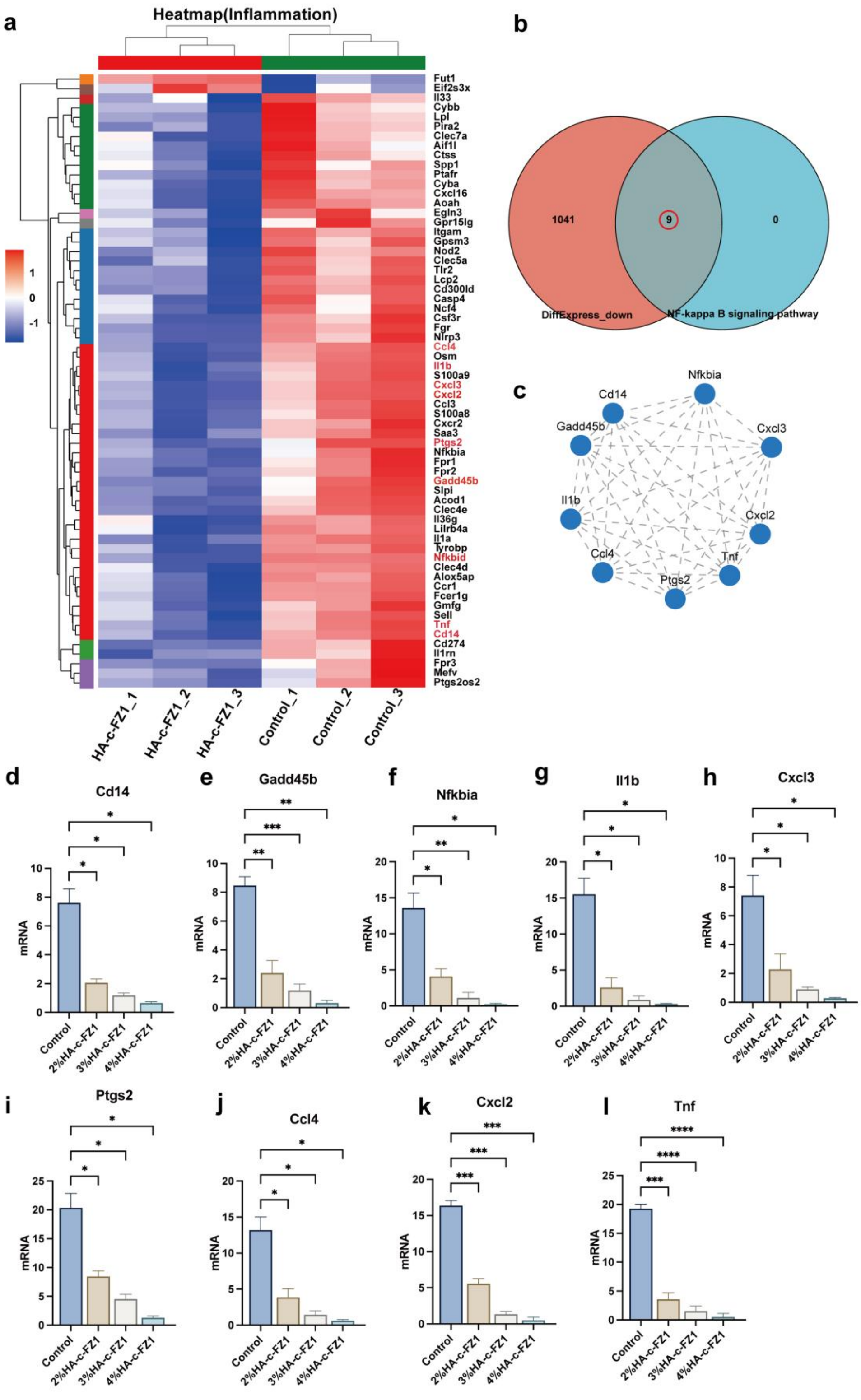

Fig. S4. Transcriptomic screening and RT-qPCR validation of NF-κB pathway-associated inflammatory genes suppressed by HA-c-FZ1 treatment. a. Heatmap of inflammation-related genes. **b**. Venn diagram showing the overlap between downregulated genes and the NF-κB

signaling pathway, identifying nine common downregulated genes (*Cd14*, *Gadd45b*, *Nfkbia*, *Il1b*, *Cxcl3*, *Ptgs2*, *Ccl4*, *Cxcl2*, *Tnf*). **c**. Protein-protein interaction (PPI) network of the nine downregulated genes. **d-l**. RT-qPCR validation of mRNA expression levels for *Cd14*, *Gadd45b*, *Nfkbia*, *Il1b*, *Cxcl3*, *Ptgs2*, *Ccl4*, *Cxcl2*, and *Tnf*; n = 3. Data are expressed as mean ± SD. Statistical significance was determined by two-tailed Student's t test or one-way ANOVA. *$p < 0.05$, **$p < 0.01$, ***$p < 0.001$, ****$p < 0.0001$.
